# Ultramicroporous Ionic Liquid-Supported Aerogel Composites

**DOI:** 10.3390/nano15070526

**Published:** 2025-03-31

**Authors:** Wenshuo Pan, Shaojuan Zeng, Jiang Chang, Guilin Li, Wei Zhang, Xiangping Zhang

**Affiliations:** 1 College of New Material and Chemical Engineering, Beijing Institute of Petrochemical Technology, Beijing 102617, China; wspan@ipe.ac.cn (W.P.); wzhang@bipt.edu.cn (W.Z.); 2 Beijing Key Laboratory of Solid State Battery and Energy Storage Process, CAS Key Laboratory of Green Process and Engineering, State Key Laboratory of Mesoscience and Engineering, Institute of Process Engineering, Chinese Academy of Sciences, Beijing 100190, China; snivy0229@163.com (J.C.); liguilin2021@ipe.ac.cn (G.L.); 3 College of Chemical Engineering and Environment, China University of Petroleum, Beijing 102249, China; xpzhang@ipe.ac.cn

**Keywords:** ionic liquids, aerogel, NH_3_ adsorption, separation, ultramicroporous

## Abstract

Recently, low-concentration ammonia (NH_3_) capture has attracted considerable attention for applications in ammonia–hydrogen fuel cells and confined spaces. The main objective of this study was to develop novel ultramicropore ionic liquid-supported aerogel composites (UILACs), designed to effectively expose the multiple hydrogen bonding sites of the ionic liquid through the constructed ultramicropore structure in order to capture and selectively separate low-concentration NH_3_. UILACs achieved a maximum NH_3_ capacity of 164.69 mg NH_3_/g absorbent at 25 °C and 0.10 MPa, which was 3.47 times higher than that of the pure aerogel. In breakthrough experiments with low NH_3_ concentrations (1000–10,000 ppm), UILACs exhibited exceptional NH_3_/H_2_ and NH_3_/N_2_ selectivity of 2460 and 10,474, respectively, at 1000 ppm NH_3_, values which are 31.5 and 22.1 times higher than the pure aerogel. These values significantly exceeded those of aerogels, owing to high hydroxyl ammonium ionic liquid (HAIL) loading, interactions between HAIL and NH_3_, and ultramicropores, as confirmed by density functional theory (DFT) calculations and isothermal analysis. Furthermore, UILACs maintained stable adsorption performance through ten adsorption–desorption cycles, demonstrating UILACs’ potentials for efficient NH_3_ capture and separation in energy applications.

## 1. Introduction

The transition to a sustainable energy future is heavily reliant on the effective harnessing and distribution of renewable energy sources. However, the variability and geographical constraints of sources like solar and wind pose significant challenges [1]. Hydrogen (H_2_) emerges as a promising energy carrier due to its high energy density and zero-emission potential, yet significant technical and economic barriers persist in its storage and transportation [2]. In this context, ammonia (NH_3_) has gained attention as a carbon-free, hydrogen-rich chemical with significant potential as both energy carrier and fuel, and theoretical H_2_ transport efficiency using NH_3_ is around 90% [3]. Although NH_3_ is a deleterious substance, it represents the least hazardous transport carrier in terms of flammability. NH_3_ can be liquefied under compression at 0.10 MPa and 25 °C, exhibiting a higher volumetric H_2_ density of approximately 0.108 kg/L, which is 1.5 times that of liquid H_2_ [4]. Additionally, its established production infrastructure and lower explosion risk compared to H_2_ further enhance its appeal [5,6]. NH_3_ as an energy storage medium, can be used in various systems such as fuel cells [3], gas turbines [7], and reciprocating engines [8] to generate power. In industrial production, NH_3_ also serves as a critical raw material for synthesizing agricultural fertilizers [9], ammonium salts [10], sulfonamides [11], polyurethanes [12], polyamides [12], and refrigerants [13].

Ammonia-based energy systems are gaining attraction as a promising pathway for sustainable energy conversion and storage [14]. In these systems, NH_3_ is catalytically decomposed into nitrogen (N_2_) and H_2_, with the latter serving as a clean fuel for power generation through electrochemical reactions [15,16]. However, NH_3_ decomposition at 500–550 °C and 0.10 MPa achieves only 99.74–99.84% conversion, leaving 800–1300 ppm residual NH_3_ [17]. Even trace NH_3_ can damage fuel cells, making the residual NH_3_ removal a critical challenge in ammonia–hydrogen fuel cell applications [18]. Hence, selective removal of residual NH_3_ is crucial to prevent fuel cell damage and comply with ISO 14687: 2019 standards, presenting a key technical challenge [19,20,21]. 

Traditional NH_3_ absorption solvents, including water and acidic solutions, face limitations in water consumption, energy requirements, and strict equipment demands. With increasing environmental concerns, the development of novel efficient absorbents has become crucial for NH_3_ purification technology. Among various alternatives, ionic liquids (ILs) exhibit exceptional gas capture capabilities due to their tunable structure [22,23]. ILs can be precisely modulated through strategic design and combination of different anions and cations, offering extensive opportunities for functionalization and diversification [24,25]. A series of ILs have been reported for NH_3_ absorption, primarily focuses on several categories: conventional ILs [26], hydroxyl ILs [22], protic ILs [27,28], and metal ILs [29,30,31]. Although these ILs exhibited significant advantages in NH_3_ absorption performance, their relatively high viscosity limited gas–liquid interface typically result in low mass transfer efficiency during gas absorption and desorption [25]. 

Combining ILs with porous materials, such as silica gel [32], molecular sieves [33,34,35,36], and activated carbon [37,38,39], had proven to be efficient in enhancing NH_3_ separation by improving mass transfer efficiency. Emerging strategies in network-structured materials design, particularly those leveraging bio-derived reagents and controlled microporous architectures, offer valuable insights for optimizing gas–solid interfaces in composite systems [40,41,42]. In 2014, Kohler et al. [43] reported IL-based composite materials prepared by immobilizing imidazolium-based ILs and metal complex films on polymer-based spherical activated carbon supports for efficient NH_3_ removal. The supported IL phase composite containing chlorometallate salts (20 vol% [C_2_C_1_Im]Cl/CuCl_2_) at 30 °C, 0.121 MPa, and 1000 ppm NH_3_ achieved 27.14 and 56.65 mg NH_3_/g adsorbent under dry and 85% relative humidity conditions, respectively. However, strong IL-NH_3_ interactions resulted in relatively low desorption efficiency of 56%. Qajar et al. [38] synthesized various carbon materials (pore sizes 0.5–12 nm) and treated them with concentrated nitric acid at 90 °C. The treatment enhanced NH_3_ capacity from 170 to 289 mg NH_3_/g absorbent at 25 °C and 0.10 MPa. However, the process reduced pore volume by 35–50%, caused material loss with extended treatment time, and required strict experimental conditions, potentially limiting practical applications. Our previous studies also demonstrated enhanced NH_3_ adsorption through IL loading on large specific surface area supports. Yu et al. [37] reported that activated carbon 980 (AC-980) loaded with 20 wt% 2-methylimidazolium bis(trifluoromethylsulfonyl)imide ([2-Mim][NTf_2_]) achieved NH_3_ capacity of 68.61 mg NH_3_/g absorbent at 25 °C and 0.10 MPa. The 30% improvement over pure AC-980 resulted from synergistic hydrogen bonding and hierarchical pore structures. Li et al. [44] developed multiproton IL (MPIL)-based molecular sieve (HZSM-5) hybrid adsorbents, which showed high NH_3_ capacity up to 140.42 mg NH_3_/g absorbent at 30 °C and 0.10 MPa, with NH_3_/CO_2_ and NH_3_/N_2_ selectivity of 41.7 and 468.0, respectively. The composite maintained stable performance through six adsorption–desorption cycles. The above studies demonstrated promising capabilities of IL-based composites for efficient NH_3_ separation through optimized structural design and functional modifications, providing potential solutions for efficient NH_3_ separation and resource utilization. However, most studies on IL composites focused on NH_3_ adsorption and separation at atmospheric pressure.

Density functional theory (DFT) has become an important tool for understanding the interactions between gases and adsorbents, providing valuable insights into adsorption mechanisms [45]. In our group’s previous study, DFT simulations were used to calculate the interaction between ILs with different hydroxyl group numbers and NH_3_. The results showed that N,N,N′,N′-tetrakis(2-hydroxyethyl)ethylenediamine trifluoromethane sulfonate ([EdteH_6_][TfO]_2_), which contains multiple protonic hydrogens and hydroxyl groups, exhibited strong interaction with NH_3_ [44]. In the DFT calculations of IL-composites, our group further investigated porous IL composites for CO_2_ adsorption and found that the interaction energy between the IL composite and CO_2_ (−45.33 kJ/mol) is significantly higher than the interaction energy between the support-CO_2_ (−15.82 kJ/mol), indicating that the introduction of ILs plays a crucial role in CO_2_ adsorption and selective separation [46].

To address the adsorption and separation of NH_3_ at extremely low concentrations, such as in ammonia–hydrogen fuel cells and specific environments, aerogels are promising supports due to their ultra-high porosity, large specific surface area, and extremely low density, which enable high IL loading while ensuring their effective dispersion [47,48]. This study developed a novel composite material, ultramicroporous ionic liquid-supported aerogel composites (UILACs), composed of hydroxyl ammonium ionic liquids (HAILs) and aerogels for efficient NH_3_ capture at extremely low concentrations. Two HAILs, N-methyldiethanolamine bis(trifluoromethanesulfonyl)imide ([MDEAH][NTf_2_]) and triethanolamine bis(trifluoromethanesulfonyl)imide ([TEAH][NTf_2_]), were designed for efficient NH_3_ capture by multiple hydrogen bonding between hydroxyl groups, proton, and NH_3_. The designed HAILs were combined with different kinds of aerogel supports with high surface area, porosity, and thermal stability to form novel UILACs, where HAILs were confined within the pore structure of aerogels, partially filling the pore space while maintaining permanent porosity. The effects of HAIL types and loading, aerogel supports, temperatures on NH_3_ capacity, and NH_3_ selectivity under binary (NH_3_/H_2_, NH_3_/N_2_) and ternary (NH_3_/H_2_/N_2_) gas mixtures were totally investigated, and ten adsorption–desorption experiments were also conducted for recyclability. NH_3_ adsorption mechanism was elucidated through pyridine IR, in situ IR, isosteric heat calculations, and density functional theory (DFT) calculations of gas interactions with HAIL, aerogel, and UILAC.

## 2. Experimental Section

### 2.1. Materials

NH_3_ (99.999 vol%), H_2_ (99.999 vol%), and N_2_ (99.999 vol%) were supplied by Beijing Yongsheng Co., Ltd. (Beijing, China). Anhydrous MgSO_4_ (99 wt%), tert-butanol (99 wt%), triethanolamine (99 wt%), N-methyldiethanolamine (99 wt%), and bis(trifluoromethanesulfonyl)imide (99 wt%) were purchased from Aladdin Industrial Corporation (Shanghai, China). Analytical-grade dichloromethane and ethanol were supplied by Beijing Chemical Works (Beijing, China). Aerogels P200 and P250F were obtained from Cabot Co., Ltd. (Shanghai, China); KM-W20 and KM-W50 were obtained from Suzhou Kangmai New Materials Co., Ltd. (Jiangsu, China); and KSL6 was sourced from IBIH Co., Ltd. (Henan, China). All the reagents were used as received, without additional purification. Detailed information about the aerogels from their suppliers is provided in Table S2.

### 2.2. Synthesis of HAILs and UILACs

Two HAILs, [MDEAH][NTf_2_] and [TEAH][NTf_2_], were synthesized via a one-step method [44]. The synthesis procedure was described using [TEAH][NTf_2_] as an example: 0.02 mol triethanolamine was dissolved in 80 mL anhydrous ethanol and placed in a round-bottom flask, and then it was cooled in an ice bath. In total, 0.02 mol bis(trifluoromethanesulfonyl)imide was added dropwise to the solution, followed by magnetic stirring for 48 h at room temperature. Anhydrous MgSO_4_ was added to the solution until no further crystallization was observed. The mixture was filtered to remove MgSO_4_, and the filtrate was washed 5 times with dichloromethane. The solvent was removed via rotary evaporation, and the product was dried in a vacuum oven at 60 °C for 48 h, yielding the HAIL [TEAH][NTf_2_].

The composites were named HAIL–aerogel-X wt%, where X represents the mass percentage of HAIL in the composite. The synthesis of [TEAH][NTf_2_]-KSL6-90 wt% was described as an example: 1.800 g [TEAH][NTf_2_] was dissolved in 20 mL tert-butanol, followed by the gradual addition of 0.200 g KSL6. The mixture was centrifuged at 8000 rpm for 10 min, and this process was repeated 5 times. The product was then lyophilized for 48 h using LGJ-10E vacuum freeze dryer to remove tert-butanol, yielding [TEAH][NTf_2_]-KSL6-90 wt%.

### 2.3. Characterization Methods of HAILs and UILACs

The structures of the synthesized HAILs were characterized by ^1^H NMR spectroscopy using a Bruker 600 MHz spectrometer (Karlsruhe, Germany) with deuterated dimethyl sulfoxide (DMSO-d_6_) as the solvent and infrared spectroscopy analysis, performed on a Nicolet 380 FTIR instrument (Thermo Fisher Scientific, Waltham, MA, USA). The spectra were collected across the wavenumber region from 400 to 4000 cm^−1^. Water content of the HAILs was determined using Karl Fischer titration (Mettler-Toledo Coulometric KF Titrator C20, Columbus, OH, USA), and all the samples contained less than 2000 ppm. 

The N_2_ adsorption–desorption isotherms of UILACs were measured using a Quantachrome Autosorb-iQ analyzer (Anton Paar, Graz, Austria). The specific surface area was calculated using the Brunauer–Emmett–Teller (BET) method in the relative pressure range of 0.05 < P/P_0_ < 0.30, while pore size distributions were determined by Barrett–Joyner–Halenda (BJH) and DFT methods [49,50]. The morphologies of UILACs were characterized by scanning electron microscopy (SEM, SU8020, Hitachi, Tokyo, Japan) and transmission electron microscopy (TEM, JEOL JEM2100, JEOL Ltd., Tokyo, Japan). Pyridine FTIR (Py-IR) spectra were recorded using a Nicolet 6070 spectrometer (Thermo Fisher Scientific, Waltham, MA, USA) in the range of 1400–1700 cm^−1^ to determine the acid sites of the UILACs. In situ FTIR spectra were collected using a Bruker INVENIO S spectrometer (Bruker, Karlsruhe, Germany) in the range of 600–4000 cm^−1^. NH_3_ adsorption was performed at 25 °C by introducing a gas mixture of 20 vol% NH_3_ balanced with N_2_ at a total flow rate of 50 mL/min. After saturation, the desorption process was carried out at 100 °C under a N_2_ flow of 50 mL/min. X-ray diffraction (XRD) patterns were collected using a Rigaku Smartlab-(9) diffractometer (Rigaku Corporation, Tokyo, Japan). The measurements were performed over the 2θ range from 10° to 60°, with a step size of 0.01°, to obtain the diffraction intensity data. Decomposition temperatures from room temperature to 800 °C were measured by TGA Q5000 (TA Instruments, New Castle, DE, USA) to determine the thermal stability at a dry N_2_ flow rate of 25 mL/min, with a heating rate of 10 °C/min.

### 2.4. NH_3_ Adsorption and Desorption Methods of HAILs and UILACs

The absorption capacity and kinetics curves of HAILs and UILACs were measured using the weighing method [51,52]. For pure HAILs, about 3.00 g sample was placed in an absorption glass vial with 1.0 cm inner diameter and preheated in a thermostatic water bath with ±0.10 °C temperature control accuracy. The NH_3_ absorption was conducted at 40 °C with a 100 mL/min NH_3_ flow rate. An electronic balance with ±0.10 mg accuracy was used to record the sample mass changes at different time intervals during NH_3_ absorption. Equilibrium was considered to be achieved when the sample mass remained constant, enabling the determination of NH_3_ absorption kinetics and final absorption capacity. For desorption, the sample was maintained at 80 °C under a N_2_ flow of 100 mL/min, and mass changes were recorded at different times. Subsequently, NH_3_ adsorption curves of UILACs were also tested at different time intervals under the same experiment conditions for comparison with pure HAILs.

Before gas adsorption experiments using a Quantachrome Instrument Autosorb-iQ-C-MP-MP, the adsorbents were firstly degassed under vacuum at 100 °C for 4 h to remove excess moisture and volatile components, and then NH_3_ adsorption isotherms of UILACs were measured. The isotherms were collected at pressures ranging from 0.001 to 0.10 MPa and temperatures from 15 to 80 °C. NH_3_ desorption was performed by heating the NH_3_-saturated UILACs at 100 °C for 4 h in a degassing system, followed by the repeated adsorption experiments.

The adsorption breakthrough curves were measured using a BSD-MAB instrument (BeiShiDe Instrument Technology). Using a binary NH_3_/H_2_ gas mixture with low-concentration of 1000 ppm NH_3_ as an example, the experimental procedure was as follows: 0.20 g sample was loaded into a breakthrough column with a 6 mm inner diameter. The sample was activated by helium gas purging at 30 mL/min flow rate at 100 °C for 4 h. After activation, the breakthrough column was cooled to room temperature and transferred to a thermostatic water bath at 25 °C. The gas flow was then switched to an NH_3_/H_2_ mixture with flow rates in the ranges from 0.02 mL/min NH_3_ to 19.98 mL/min H_2_, and the adsorption breakthrough experiment was conducted until sample saturation. The total flow rate of the mixed gas was maintained at 20 mL/min for all the breakthrough experiments.

### 2.5. Simulated Calculations

In this study, all DFT calculations were conducted using Gaussian16 software package, Revision C.01. Geometries of all structures were optimized at the B3LYP-D3(BJ)/def2-SVP level, where the inclusion of empirical dispersion corrections with Becke–Johnson damping ensured accurate characterization of van der Waals interactions critical for gas adsorption systems. The single point calculations were performed using the def2-TZVP basis set, and the counterpoise corrections were used when computing the complexation energy to avoid the basis set superposition errors. The calculation method for the interaction energy (ΔE) is given by Equation (1):

∆E = E_P_ − E_R_ (1)

where E_p_ represents the energy of the product (kJ/mol), E_R_ represents the energy of the reactant (kJ/mol), and ΔE represents the energy difference between the product and the reactant (kJ/mol).

## 3. Results and Discussion

### 3.1. Characterizations of HAILs and UILACs

**Figure 1:**
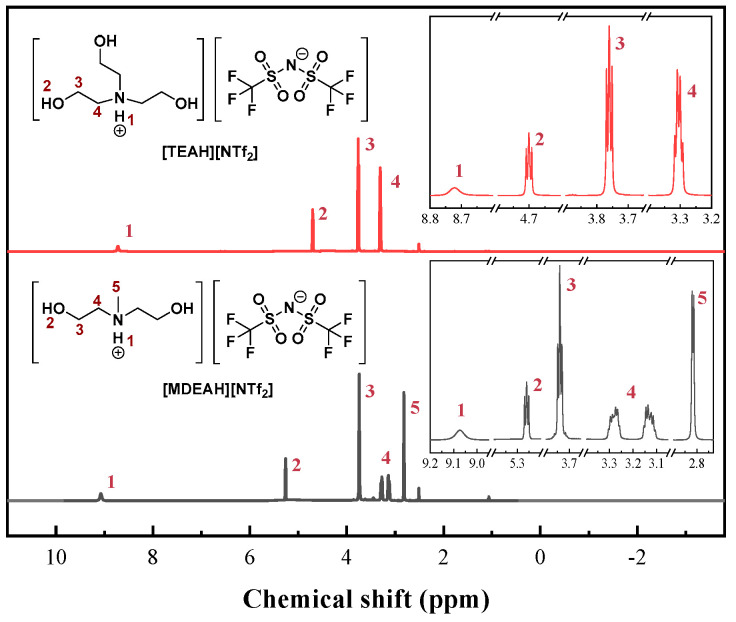
^1^H NMR spectra and structure of two HAILs.

The structures of the synthesized HAILs were verified using ^1^H NMR spectroscopy, as shown in Figure 1. The ^1^H NMR spectrum of [TEAH][NTf_2_] showed characteristic signals at δ = 8.72 (s, 1H, -NH), 4.57 (t, 3H, -OH), 3.76 (m, 6H, -CH_2_OH), and 3.30 ppm (t, 6H, -NCH_2_-). The integration ratios of these signals (1:3:6:6) were consistent with the expected molecular structure [53]. Similar spectral analysis confirmed the successful synthesis of [MDEAH][NTf_2_].

**Figure 2:**
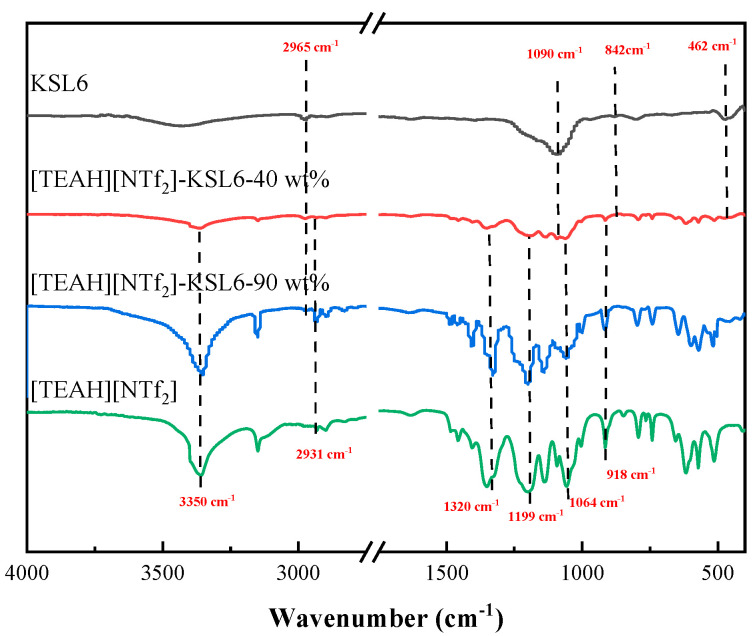
FTIR spectra of [TEAH][NTf_2_], KSL6, and [TEAH][NTf_2_]-KSL6 composites.

The FTIR spectra of the HAILs, aerogels, and UILACs are also characterized in Figure 2. For the HAILs, the peak at 3350 cm^−1^ was attributed to the stretching vibrations of -OH and -NH groups. The peak at 2931 cm^−1^ corresponded to the stretching vibrations of -CH, the 1400–1500 cm^−1^ range corresponded to the bending or flexing vibrations of -CH_2_, and absorption peak near 918 cm^−1^ was attributed to the C-N stretching vibration of the cation in the cation. The FTIR spectra of [NTf_2_]^-^ anion showed that the peak of wavenumber at 1064 cm^−1^ was assigned to S-N-S stretching vibration. The peak at 1199 cm^−1^ originated from the asymmetric stretching vibration of C-F [36,54,55]. For the pure aerogels, the peaks at 1090, 842, and 462 cm^−1^ corresponded to the asymmetric and symmetric stretching vibrations of Si-O-Si, respectively. The peak at 2965 cm^−1^ was due to the C-H asymmetric stretching vibration of the methyl (-CH_3_) group [49]. Compared with the FTIR of pure HAILs and aerogels, [TEAH][NTf_2_]-KSL6-40 wt% with HAIL loading exhibited the characteristic peaks of both aerogel and HAIL. As the HAIL loading increased to 90 wt%, [TEAH][NTf_2_]-KSL6-90 wt% predominantly showed the characteristic peaks of HAIL, with the aerogel’s characteristic peak observed only at 2965 cm^−1^. This was likely due to the overlapping between the Si-O-Si framework vibrations of aerogels and the C-F and S-N-S functional group vibrations of HAIL, and the extremely high loading of HAIL could cover aerogel surface, thereby masking the characteristic signals of most surface groups of aerogel [48]. The FTIR spectra of P200, P250F, [TEAH][NTf_2_]-P200-90 wt%, and [TEAH][NTf_2_]-P250F-90 wt% are shown in Figure S1.

In addition, the XRD results revealed that the aerogels exhibited typical characteristics of amorphous silica, as shown in Figure S2. A broad diffraction peak was observed at 2θ ≈ 22°, which is characteristic of amorphous SiO_2_. After loading HAILs, the main diffraction peak shifted to a lower angle (approximately 17°), while UILACs maintained their amorphous structural features, indicating that the incorporation of HAILs did not alter the fundamental structures of the aerogel. This peak shift phenomenon can be primarily attributed to the penetration of HAIL molecules into the pore channels of the aerogel. Insertion of HAIL led to the expansion of the SiO_2_ network structure, resulting in an increase in interplanar spacing (d-value). According to Bragg’s equation (2d·sinθ = nλ), an increase in d-value leads to a decrease in diffraction angle, θ, thus causing the diffraction peak to shift toward lower angles [56,57]. 

**Figure 3:**
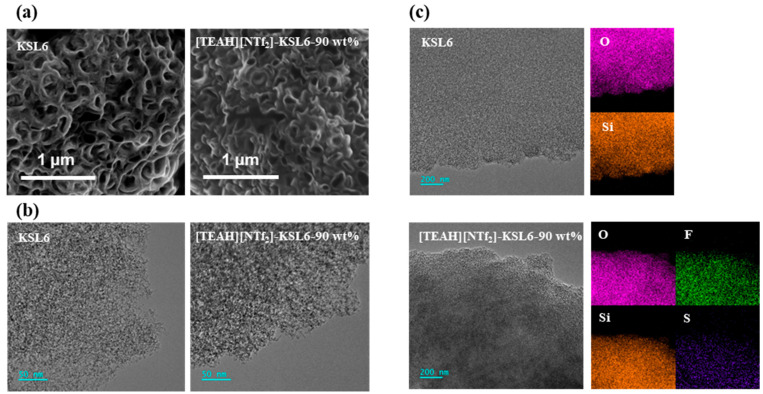
(**a**) SEM images, (**b**) TEM images, and (**c**) mapping images of KSL6 and [TEAH][NTf_2_]-KSL6-90 wt%.

SEM and TEM observations revealed that the incorporation of [TEAH][NTf_2_] did not significantly alter the porous network structure of the aerogel. The pore structure on surface of [TEAH][NTf_2_]-KSL6-90 wt% was well preserved after HAIL loading, as demonstrated in Figure 3a,b. Elemental mapping analysis further confirmed the uniform distribution of HAIL on KSL6, where Si and O elements originate from the silica framework, while F and S elements derive from [NTf_2_]^−^ of HAIL, as presented in Figure 3c. SEM, TEM, and mapping images of P200, P250F, [TEAH][NTf_2_]-P200-90 wt%, and [TEAH][NTf_2_]-P250F-90 wt% are shown in Figures S3 and S4.

### 3.2. Properties of HAILs and UILACs

The thermogravimetric analysis (TGA) results of pure HAILs, [TEAH][NTf_2_] and [MDEAH][NTf_2_]; and aerogels P200, P250F, KSL6, and UILACs are given in Figure S5 and Table 1. The two HAILs showed an onset decomposition temperature at 239.32 °C. For the aerogels, thermal decomposition temperatures were 580.77 °C for P200, 591.60 °C for P250F, and 506.92 °C for KSL6. For UILACs, their thermal stability was intermediate between HAIL and aerogels. The thermal decomposition temperatures of [TEAH][NTf_2_]-P200-90 wt%, [TEAH][NTf_2_]-P250F-90 wt%, and [TEAH][NTf_2_]-KSL6-90 wt% were 268.92, 270.11, and 258.30 °C, respectively. These results indicated that HAIL was successfully incorporated onto the aerogel, and its introduction significantly influenced the thermal stability of the composites. 

**Figure 4:**
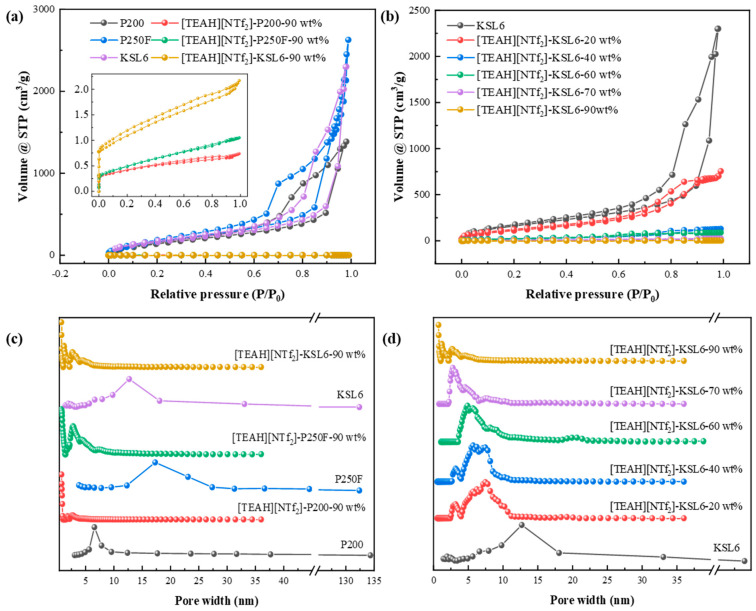
N_2_ adsorption–desorption isotherms of (**a**) aerogels and UILACs; and (**b**) [TEAH][NTf_2_]-KSL6 composites. Pore size distributions of (**c**) aerogels and UILACs; and (**d**) [TEAH][NTf_2_]-KSL6 composites.

The N_2_ adsorption isotherms of pure aerogels in Figure 4a showed type IV characteristics, with an H3 hysteresis loop, and no distinct adsorption plateau. The absence of adsorption saturation at high relative pressures indicated an irregular pore structure, including plate-like slits, cracks, and wedge-shaped formations [58,59]. The aerogels exhibited pore sizes primarily in the range of 325 nm, according to the BJH model and characteristics of wide distribution mesoporous aerogels, as represented in Figure 4c. In contrast, the UILACs with 90 wt% HAIL loading displayed a hybrid type I and II isotherm with an H4 hysteresis loop, indicating the presence of microspores and mesopores, with pore sizes primarily in the range of 0.7–5 nm, according to the DFT model [60].

In addition, the effect of HAIL loadings from 0 to 90 wt% on N_2_ adsorption isotherms in Figure 4b shows that with the increase in HAIL loading, the hysteresis loop of [TEAH][NTf_2_]-KSL6 composites gradually becomes narrow, and the pore sizes decrease in [TEAH][NTf_2_]-KSL6 composites gradually, which were 12.694, 2.187, 5.689, 4.887, 2.769, and 0.718 nm when HAIL loadings were 0, 20, 40, 60, 80, and 90 wt%, respectively, as shown in Figure 4d. Moreover, the ultramicroporous structure appears only at 90 wt% HAIL loading. Thermal stability and pore structures of aerogels and UILACs are summarized in Table 1. The N_2_ adsorption–desorption isotherms and pore size distributions of [TEAH][NTf_2_]-P200 composites and [TEAH][NTf_2_]-P250F composites are shown in Figure S6.

### 3.3. NH_3_ Adsorption Performance of UILACs

#### 3.3.1. Effect of HAILs and Aerogel Supports

**Figure 5:**
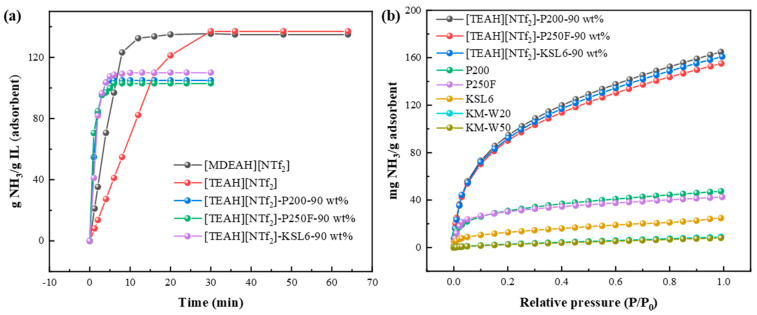
(**a**) NH_3_ absorption/adsorption curves of HAILs and UILACs at 40 °C and 0.10 MPa. (**b**) NH_3_ adsorption isotherms of aerogels and UILACs at 25 °C and 0.10 MPa.

The NH_3_ absorption curves of HAILs were first determined using the gravimetric method, and the results are shown in Figure 5a. At 40 °C and 0.10 MPa, NH_3_ absorption capacities of [MDEAH][NTf_2_] with one proton and two hydroxyl groups and [TEAH][NTf_2_] with one proton and three hydroxyl groups were 0.134 and 0.137 mg NH_3_/g IL (corresponding to 3.18 and 3.45 mol NH_3_/mol IL), respectively, demonstrating that the NH_3_ absorption capacity of HAILs exhibits a positive correlation with the number of hydrogen bond donors. However, the introduction of hydroxyl groups also led to increased viscosity, which affected the equilibrium time of NH_3_ absorption. The viscosities of [MDEAH][NTf_2_] and [TEAH][NTf_2_] were 88.795 and 190.06 mPa·s at 40 °C, corresponding to the equilibrium times of 20 and 30 min, respectively. Based on these results, [TEAH][NTf_2_] was selected for subsequent optimization experiments.

For aerogels, the pore structures and thermal decomposition temperature of five aerogels are summarized in Table 1. Compared with two other aerogels, KM-W20 and KM-W50, the aerogels P200, P250F, and KSL6 showed higher specific surface areas over 500 m^2^/g and high thermal decomposition temperatures above 500 °C. Meanwhile, P200, P250F, and KSL6 also exhibited higher NH_3_ capacity of 47.429, 42.537, and 24.849 mg NH_3_/g adsorbent at 25 °C and 0.10 MPa, respectively, as shown in Figure 5b. Therefore, considering their comprehensive performance, P200, P250F, and KSL6 were selected as supports for further investigation.

After optimization of HAIL and aerogels, NH_3_ adsorption curves of the synthesized UILACs were obtained as shown in Figure 5a. Compared to pure HAIL [TEAH][NTf_2_], which required 30 min to reach absorption equilibrium, UILACs achieved equilibrium within 6 min, indicating that the incorporation of HAILs into aerogels can effectively improve the mass transfer rate and shorten the equilibrium time due to the high viscosity of HAILs. Meanwhile, the UILACs showed significantly enhanced NH_3_ adsorption performance compared to pure aerogels. At 0.10 MPa, the NH_3_ capacity of [TEAH][NTf_2_]-P200-90 wt%, [TEAH][NTf_2_]-P250F-90 wt%, and [TEAH][NTf_2_]-KSL6-90 wt% reached 164.69, 155.09, and 160.09 mg NH_3_/g adsorbent, respectively. At 0.001 MPa, the NH_3_ capacity of P200, P250F, and KSL6 was 4.84, 4.80, and 2.56 mg NH_3_/g adsorbent, respectively, while the NH_3_ capacity of [TEAH][NTf_2_]-P200-90 wt%, [TEAH][NTf_2_]-P250F-90 wt%, and [TEAH][NTf_2_]-KSL6-90 wt% increased to 7.33, 6.10, and 8.29 mg NH_3_/g adsorbent, respectively. Among them, [TEAH][NTf_2_]-KSL6-90 wt% exhibited the highest NH_3_ capacity at low NH_3_ partial pressures. After HAIL incorporation, the superior surface area and pore volume of [TEAH][NTf_2_]-KSL6-90 wt%, with both specific surface area and pore volume higher than the other two composites, contributed to its enhanced low NH_3_ partial pressures capture performance.

Based on the above studies, [TEAH][NTf_2_], which contains protonated hydrogen and polyhydroxy groups, was selected to ensure high NH_3_ absorption. Among the five kinds of aerogels, P200, P250F, and KSL6 exhibited better NH_3_ adsorption performance due to their higher specific surface area and larger pore volume, and they were chosen as the supports for further research. When HAILs were incorporated into the aerogels, the UILACs showed higher NH_3_ capacity compared to pure aerogels, and faster equilibrium time compared to HAILs.

#### 3.3.2. Effect of HAIL Loading and Temperature on NH_3_ Adsorption

**Figure 6:**
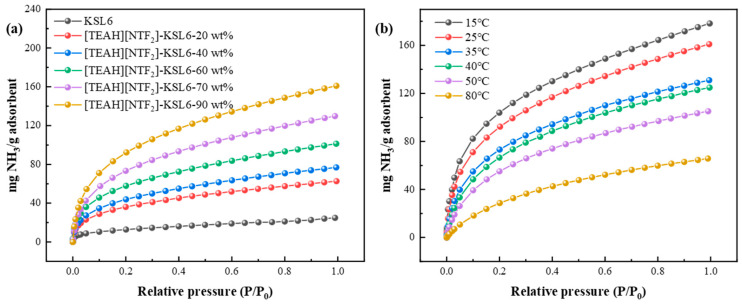
NH_3_ capacity of (**a**) KSL6 with different HAIL loadings and (**b**) [TEAH][NTf_2_]-KSL6-90 wt% at different temperatures.

Based on the above studies, the HAIL [TEAH][NTf_2_] was supported onto the three aerogels, P200, P250F, and KSL6, and the effect of HAIL loadings on NH_3_ adsorption was also studied. It was found that loadings of HAIL significantly affect NH_3_ adsorption performance. NH_3_ capacity increased with the increase of HAIL loadings from 20 to 90 wt%, as shown in Figure 6a. However, excessive HAIL loading is not beneficial, as it is crucial to maintain the structural integrity of the aerogel and prevent complete pore blockage. The results demonstrated that UILACs exhibited the highest NH_3_ capacity when the optimal HAIL loading is 90 wt%, as shown in Figure 6a. Notably, when loadings further increased to 91 wt%, the excessive HAIL completely blocked aerogel pores, exceeding the maximum loading and resulting in a highly viscous composites, as shown in Figures S7–S9.

Temperature was a critical factor influencing NH_3_ adsorption performance. NH_3_ capacity of UILACs was evaluated at different temperatures (15–80 °C) under 0–0.10 MPa. The experimental results revealed a significant inverse relationship between temperature and NH_3_ capacity. For example, [TEAH][NTf_2_]-KSL6-90 wt% exhibited the highest capacity of 160.09 mg NH_3_/g adsorbent at 25 °C, which decreased substantially to 65.76 mg NH_3_/g adsorbent at 80 °C, as observed in Figure 6b. It can be inferred that higher temperatures are more favorable for NH_3_ desorption. The effects of loading and temperature on [TEAH][NTf_2_]-P200 composites and [TEAH][NTf_2_]-P250F composites are shown in Figures S10 and S11.

#### 3.3.3. Breakthrough Experiments of Binary and Ternary Mixed Gases

To evaluate the potential application of UILACs in selective NH_3_ capture, the breakthrough experiments were conducted at 25 °C and 0.10 MPa using mixed gases, including binary gas mixtures of NH_3_/H_2_ and NH_3_/N_2_ with NH_3_ concentrations of 1000, 4000, and 10,000 ppm and a ternary gas mixture of NH_3_/H_2_/N_2_ (0.1 vol%/75.0 vol%/24.9 vol%) to study low-concentration NH_3_ adsorption performance. The detailed experimental data are presented in Tables S4–S6.

**Figure 7:**
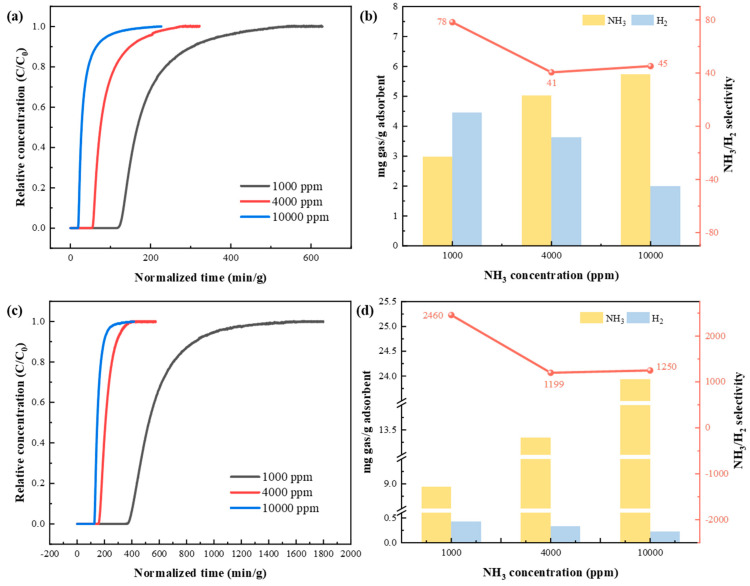
Breakthrough experiments of NH_3_/H_2_ binary mixtures at different NH_3_ concentrations: breakthrough curves for (**a**) KSL6 and (**c**) [TEAH][NTf_2_]-KSL6-90 wt%. NH_3_/H_2_ capacity and selectivity for (**b**) KSL6 and (**d**) [TEAH][NTf_2_]-KSL6-90 wt%.

For NH_3_/H_2_ separation under binary gas mixtures of NH_3_/H_2_ with NH_3_ concentrations of 1000, 4000, and 10,000 ppm, NH_3_ and H_2_ adsorption performance of KSL6 and [TEAH][NTf_2_]-KSL6-90 wt% was evaluated, as shown in Figure 7. The results demonstrate that as the NH_3_ concentration increased from 1000 to 10,000 ppm, the NH_3_ breakthrough time of both aerogels and UILACs decreased, and the adsorption rate accelerated. Moreover, compared with pure aerogels, the breakthrough time of UILACs was significantly longer at the same NH_3_ concentration, resulting in a higher NH_3_ capacity for UILACs. For instance, under a NH_3_ concentration of 1000 ppm, the breakthrough time of KSL6 was 148.149 min/g, whereas that of [TEAH][NTf_2_]-KSL6-90 wt% extended to 374.059 min/g, which is 2.52 times longer than the former, indicating the role of HAILs in NH_3_ adsorption. Therefore, the NH_3_ capacity of [TEAH][NTf_2_]-KSL6-90 wt% increased significantly to 8.937 mg NH_3_/g adsorbent compared with that of KSL6 (2.972 mg NH_3_/g adsorbent) under a NH_3_ concentration of 1000 ppm, and the NH_3_ capacity increased to 23.931 mg NH_3_/g adsorbent as the concentration of NH_3_ increased to 10,000 ppm. 

On the contrary, as the NH_3_ concentration increased, the H_2_ capacity of both aerogels and UILACs decreased. At the 1000 ppm NH_3_ concentration, the H_2_ capacity of KSL6 was 4.464 mg H_2_/g adsorbent, while that of [TEAH][NTf_2_]-KSL6-90 wt% was 0.427 mg H_2_/g adsorbent. When the NH_3_ concentration increased to 10,000 ppm, the H_2_ capacity of KSL6 dropped to 1.991 mg H_2_/g adsorbent, and that of [TEAH][NTf_2_]-KSL6-90 wt% was 0.223 mg H_2_/g adsorbent. KSL6 exhibited a high H_2_ capacity, while the H_2_ uptake of [TEAH][NTf_2_]-KSL6-90 wt% was negligible. The difference was attributed to the significant reduction in specific surface area and pore diameter of UILACs after HAIL loading, which decreased H_2_ adsorption, so selective NH_3_ separation of NH_3_/H_2_ mixture was achieved by UILACs. Simultaneously, the NH_3_/H_2_ selectivity of KSL6 was 78, while [TEAH][NTf_2_]-KSL6-90 wt% exhibited a dramatically improved selectivity of 2459, 31.5 times higher than KSL6. The selectivity was calculated using the ideal adsorbed solution theory equation, as shown in Equation (S1). The breakthrough curves of NH_3_/H_2_ binary mixtures for [TEAH][NTf_2_]-P200-90 wt% and [TEAH][NTf_2_]-P250F-90 wt% are shown in Figures S12 and S13.

**Figure 8:**
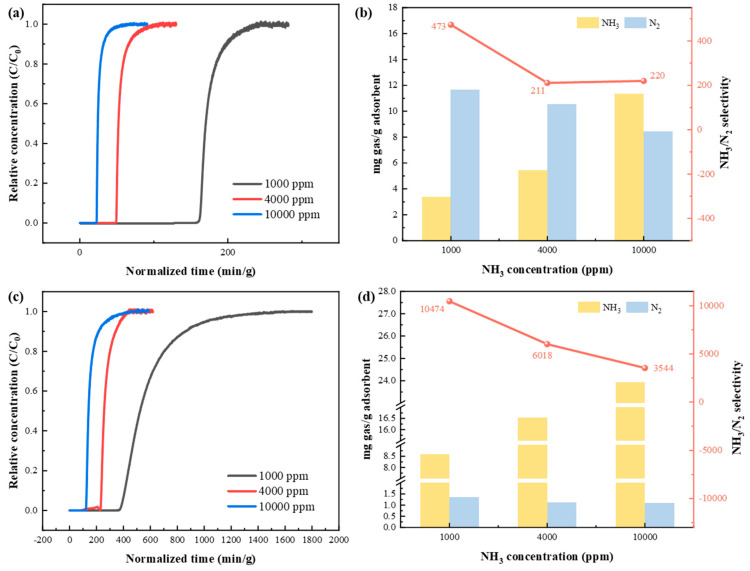
Breakthrough experiments of NH_3_/N_2_ binary mixtures at different NH_3_ concentrations: breakthrough curves for (**a**) KSL6 and (**c**) [TEAH][NTf_2_]-KSL6-90 wt%. NH_3_/N_2_ capacity and selectivity for (**b**) KSL6 and (**d**) [TEAH][NTf_2_]-KSL6-90 wt%.

Furthermore, breakthrough experiments of KSL6 and [TEAH][NTf_2_]-KSL6-90 wt% were conducted in NH_3_/N_2_ binary gas mixtures, as shown in Figure 8. Experimental results demonstrated that the separation tests of NH_3_/N_2_ and NH_3_/H_2_ exhibit similar patterns: both breakthrough time and capacity showed similar trends with varying NH_3_ concentrations. At a 1000 ppm NH_3_ concentration, KSL6 exhibited a breakthrough time of 160.486 min/g and a capacity of 3.357 mg NH_3_/g adsorbent, while [TEAH][NTf_2_]-KSL6-90 wt% demonstrated a superior performance under same conditions, achieving a breakthrough time of 374.567 min/g and a capacity of 8.587 mg NH_3_/g adsorbent. As the NH_3_ concentration increased, the N_2_ capacity of both aerogels and UILACs decreased. At a 1000 ppm NH_3_ concentration, the N_2_ capacity of KSL6 was 11.674 mg N_2_/g adsorbent, while that of [TEAH][NTf_2_]-KSL6-90 wt% was 1.349 mg N_2_/g adsorbent. When the NH_3_ concentration increased to 10,000 ppm, the N_2_ capacity of KSL6 dropped to 8.419 mg N_2_/g adsorbent, and that of [TEAH][NTf_2_]-KSL6-90 wt% was 1.101 mg N_2_/g adsorbent. Notably, [TEAH][NTf_2_]-KSL6-90 wt% displayed remarkably high NH_3_/N_2_ selectivity at 1000 ppm, reaching 10,474, which is 22.1 times higher than that of KSL6. The breakthrough curves of NH_3_/N_2_ binary mixtures for [TEAH][NTf_2_]-P200-90 wt% and [TEAH][NTf_2_]-P250F-90 wt% are shown in Figures S14 and S15.

**Figure 9:**
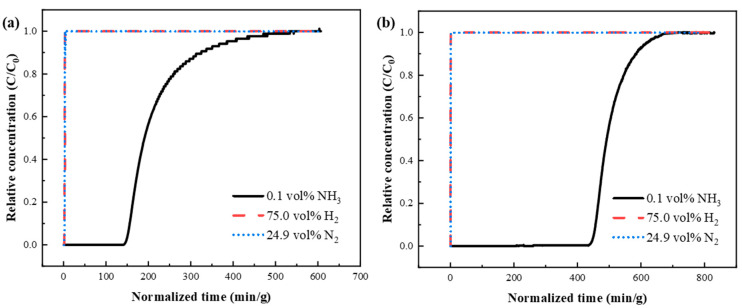
Breakthrough curves for NH_3_/N_2_/H_2_ (0.1 vol%/24.9 vol%/75.0 vol%) ternary mixtures through different composites: (**a**) KSL6 and (**b**) [TEAH][NTf_2_]-KSL6-90 wt%.

Subsequently, the breakthrough performance of KSL6 and [TEAH][NTf_2_]-KSL6-90 wt% was tested in a ternary gas mixture of NH_3_/N_2_/H_2_ (0.1 vol%/24.9 vol%/75.0 vol%), as shown in Figure 9. In the ternary system, UILACs exhibited significantly increased NH_3_ capacity and breakthrough time compared to aerogels, while the adsorption capacities for H_2_ and N_2_ were reduced. Taking KSL6 and [TEAH][NTf_2_]-KSL6-90 wt% as examples, the breakthrough time of [TEAH][NTf_2_]-KSL6-90 wt% was 444.035 min/g, three times longer than that of KSL6, which was 146.337 min/g. The NH_3_ capacity of KSL6 was 3.263 mg NH_3_/g adsorbent, whereas [TEAH][NTf_2_]-KSL6-90 wt% achieved an enhanced capacity of 7.666 mg NH_3_/g absorbent. In summary, the introduction of UILACs significantly enhanced NH_3_ adsorption performance and selectivity in low-concentration NH_3_ capture. The breakthrough curves of NH_3_/N_2_/H_2_ ternary mixtures for [TEAH][NTf_2_]-P200-90 wt% and [TEAH][NTf_2_]-P250F-90 wt% are shown in Figures S16 and S17. In breakthrough experiments under the same NH_3_ concentration, [TEAH][NTf_2_]-KSL6-90 wt% exhibited superior performance, with a longer breakthrough time, higher NH_3_ capacity, and better selectivity compared to [TEAH][NTf_2_]-P200-90 wt% and [TEAH][NTf_2_]-P250F-90 wt%. This trend aligns with the results obtained from static adsorption experiments.

For the outlet gas after breakthrough adsorption, the NH_3_ concentration was measured using the CZY50 long-tube gas detector from Yinuo Technology Co., Ltd., Shenzhen, China. A 100 mL sample of the outlet gas was drawn and passed through the gas detection tube, with the NH_3_ concentration determined by observing the color change in the detection tube. The NH_3_ concentration in the tail gas after breakthrough with the ternary components was approximately 10 ppm, as shown in Figure S18.

Through the breakthrough experiments of different components and concentrations, it can be concluded that the selective separation performance of NH_3_ significantly increased due to the incorporation of HAIL. Compared to pure aerogels, the UILACs exhibited longer breakthrough times and higher NH_3_ adsorption capacities. Under 1000 ppm NH_3_ concentration, the NH_3_/H₂ selectivity was increased 9.6–31.5 times, while the NH_3_/N₂ selectivity was increased 13.6–22.1 times. Furthermore, after breakthrough adsorption of the NH_3_/N_2_/H_2_ ternary mixture, the outlet concentration of NH_3_ using the UILACs could be reduced to around 10 ppm.

#### 3.3.4. Regeneration and Recycling of UILACs

**Figure 10:**
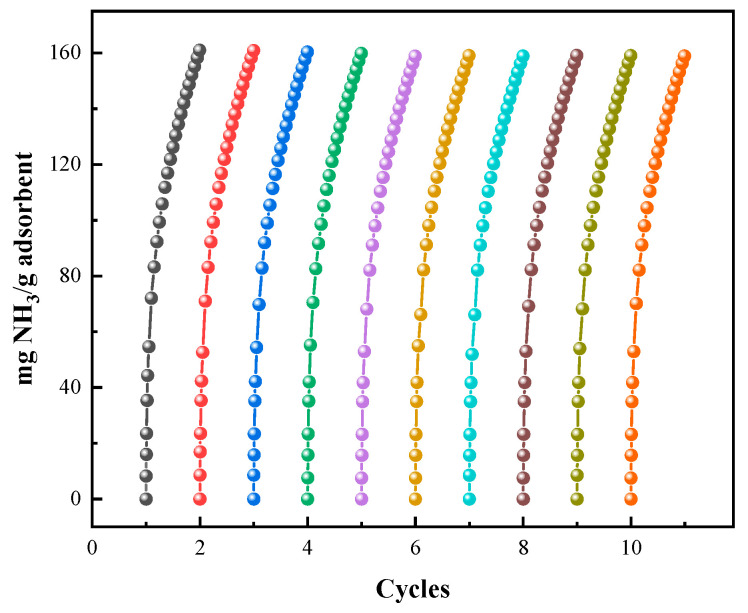
NH_3_ capacity of [TEAH][NTf_2_]-KSL6-90 wt% after 10 cycles.

Regeneration ability is a key factor in industrial applications. Therefore, cyclic adsorption–desorption tests were conducted on UILACs. The regeneration of UILACs was performed under vacuum at 100 °C for approximately 6 h. NH_3_ adsorption isotherms of UILACs at 25 °C and 0.10 MPa are shown in Figure 10. After 10 adsorption–desorption cycles, the NH_3_ capacity of the UILACs remains stable, with a decrease in adsorption capacity of less than 3%, demonstrating good stability and recoverability, making them suitable for repeated use in practical applications. The NH_3_ capacity of [TEAH][NTf_2_]-P200-90 wt% and [TEAH][NTf_2_]-P250F-90 wt% after 10 cycles is shown in Figure S19.

**Figure 11:**
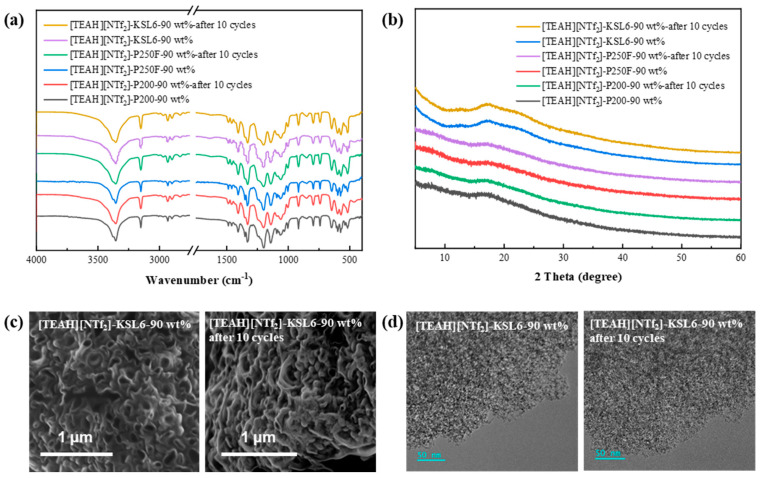
Characterization of (**a**) FTIR spectra, (**b**) XRD patterns, (**c**) SEM images, and (**d**) TEM images before and after 10 cycles.

To evaluate the structural stability of UILACs before and after cycling, multiple characterization techniques were employed. The FTIR spectral exhibited nearly identical characteristic peak positions and intensities before and after cycling, indicating stable chemical structure, as shown in Figure 11. XRD patterns revealed no significant changes in structure, while SEM and TEM images further confirmed that the composites maintained their original morphological features and microstructure throughout the cycling process. These characterization results collectively demonstrate the excellent structural stability and recyclability of the UILACs. SEM and TEM images of [TEAH][NTf_2_]-P200-90 wt% after 10 cycles and [TEAH][NTf_2_]-P250F-90 wt% after 10 cycles are shown in Figure S20.

#### 3.3.5. Comparison of NH_3_ Adsorption Performance

To evaluate the application potential of UILACs, their NH_3_ adsorption performance was compared with other porous composites reported in the literature, summarized in Table S6. Although porous IL composites have attracted considerable attention in NH_3_ adsorption in recent years, related studies remain limited. The comparison revealed that UILACs exhibited not only excellent NH_3_/H_2_ and NH_3_/N_2_ selectivity but also high NH_3_ capacity. Furthermore, the aerogel carrier, benefiting from its mature industrial production technology and relatively low cost, could significantly enhance the practical application value of UILACs. Based on these characteristics, UILACs demonstrated promising potential as efficient trace NH_3_ separation composites.

### 3.4. NH_3_ Adsorption Mechanisms by UILACs

**Figure 12:**
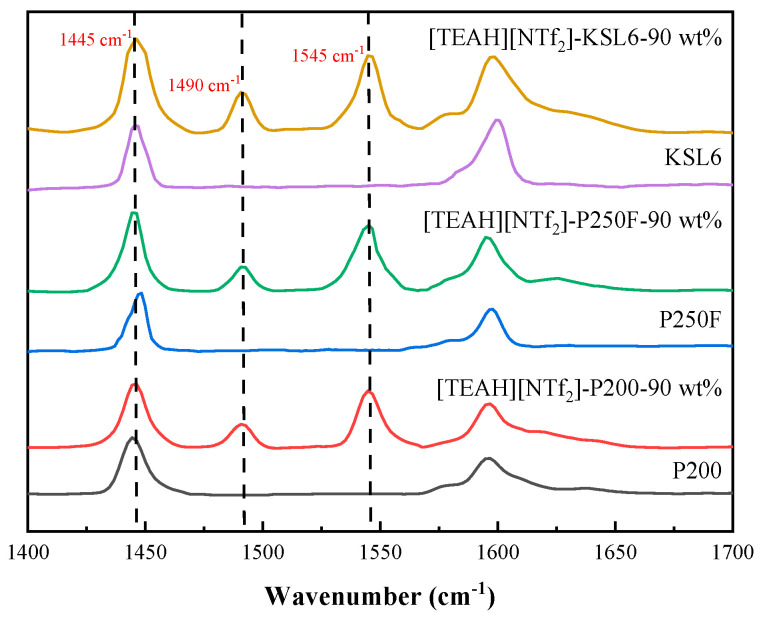
Py-IR spectra of aerogels and UILACs.

Acidity analysis of aerogels and UILACs was conducted using pyridine infrared spectroscopy, as presented in Figure 12. The characterization results revealed that the aerogels contained negligible Brønsted acid (B acid) sites but exhibited a high concentration of Lewis acid (L acid) sites. This Lewis acidity can be attributed to the silicon atoms in the aerogel framework acting as electron pair acceptors. The comparison of the properties of three aerogels in Table 2 demonstrates a positive correlation between NH_3_ capacity and L acid site content. Upon incorporation of HAILs, the B acid site content increased significantly, and the UILACs showed a positive correlation between NH_3_ capacity and B acid site content. The positive correlation between acidic sites and NH_3_ capacity can be explained by different interaction mechanisms. For L acid sites, the silicon atoms in the aerogel framework act as electron-pair acceptors, forming coordination bonds with the lone pair electrons of NH_3_ molecules. For B acid sites, functional groups in HAILs, such as hydroxyl (-OH) and amino (-NH) groups, serve as proton donors, forming stable ammonium ions (NH_4_^+^) with NH_3_ through acid–base neutralization reactions. The synergistic effect of these two types of acidic sites significantly enhanced the NH_3_ capture performance of UILACs. Furthermore, the linear relationship between acidic site content and NH_3_ capacity confirmed that both B acid and L acid sites serve as effective binding sites for NH_3_ molecules.

**Figure 13:**
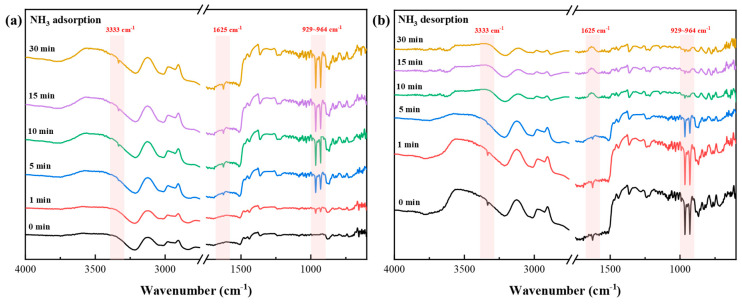
In situ FTIR spectra of (**a**) NH_3_ adsorption and (**b**) desorption on [TEAH][NTf_2_]-KSL6-90 wt%.

Meanwhile, the in situ IR analysis of [TEAH][NTf_2_]-KSL6-90 wt% revealed that NH_3_ adsorption reached saturation within approximately 10 min. The N-H stretching vibration peaks of NH_3_ are observed at 929–964 cm^−1^, while the stretching vibration peaks of N-H bond in NH_3_ and NH_4_^+^ appeared at 3333 cm^−1^ and 1626 cm^−1^, respectively, as depicted in Figure 13. The intensity of these peaks increases significantly with adsorption time, indicating that [TEAH][NTf_2_]-KSL6-90 wt% interacts with NH_3_ via H_2_ bonding. During the desorption process, complete desorption of NH_3_ was achieved within approximately 10 min, and the aforementioned peaks disappeared entirely. This demonstrated that [TEAH][NTf_2_]-KSL6-90 wt% can completely desorb NH_3_ and that the adsorption–desorption process was cyclically regenerable.

The Freundlich model, as described in Equation (S2), was employed for data fitting, which assumes adsorption occurs on heterogeneous surfaces with multilayer adsorption. The fitting results in Table S3 show R² values greater than 0.99, confirming that the adsorption behavior aligns well with heterogeneous surface characteristics. The isosteric heat of adsorption was calculated using the Clausius–Clapeyron equation, as shown in Equation (S3), based on the P and T values obtained from Freundlich model fitting at different NH_3_ capacities.

**Figure 14:**
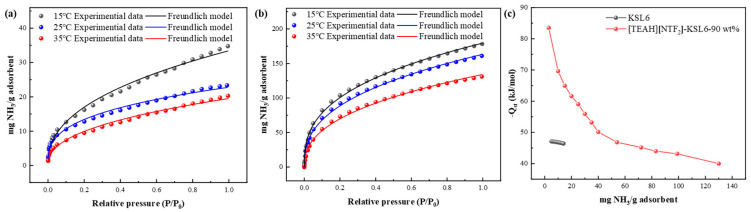
NH_3_ adsorption data and Freundlich fitting curves at 15, 25, and 35 °C for (**a**) KSL6 and (**b**) [TEAH][NTf_2_]-KSL6-90 wt%. (**c**) Isosteric adsorption heat of KSL6 and [TEAH][NTf_2_]-KSL6-90 wt%.

The adsorption heat curves, exhibited in Figure 14, revealed that the initial isosteric heat of adsorption for KSL6 was approximately −45 kJ/mol, indicating its inherent physical adsorption. After loading [TEAH][NTf_2_], the initial isosteric heat of adsorption increased significantly, with an initial adsorption heat of −84 kJ/mol, which can be attributed to the introduction of additional active sites by HAIL on the aerogel surface, enhancing the chemical interaction with NH_3_. [TEAH][NTf_2_]-KSL6-90 wt% exhibited notably higher adsorption heat at a low NH_3_ capacity, but the adsorption heat gradually decreased and plateaued as the capacity increased. This exponential decay characteristic reflects the heterogeneity of adsorption sites on the UILACs surface [61]. As the most active sites were preferentially occupied, the activity of remaining sites progressively decreased, leading to a reduction in adsorption heat with increasing NH_3_ capacity. These results confirm that incorporation of HAIL substantially enhanced the chemical capacity of the UILACs toward NH_3_.

**Figure 15:**
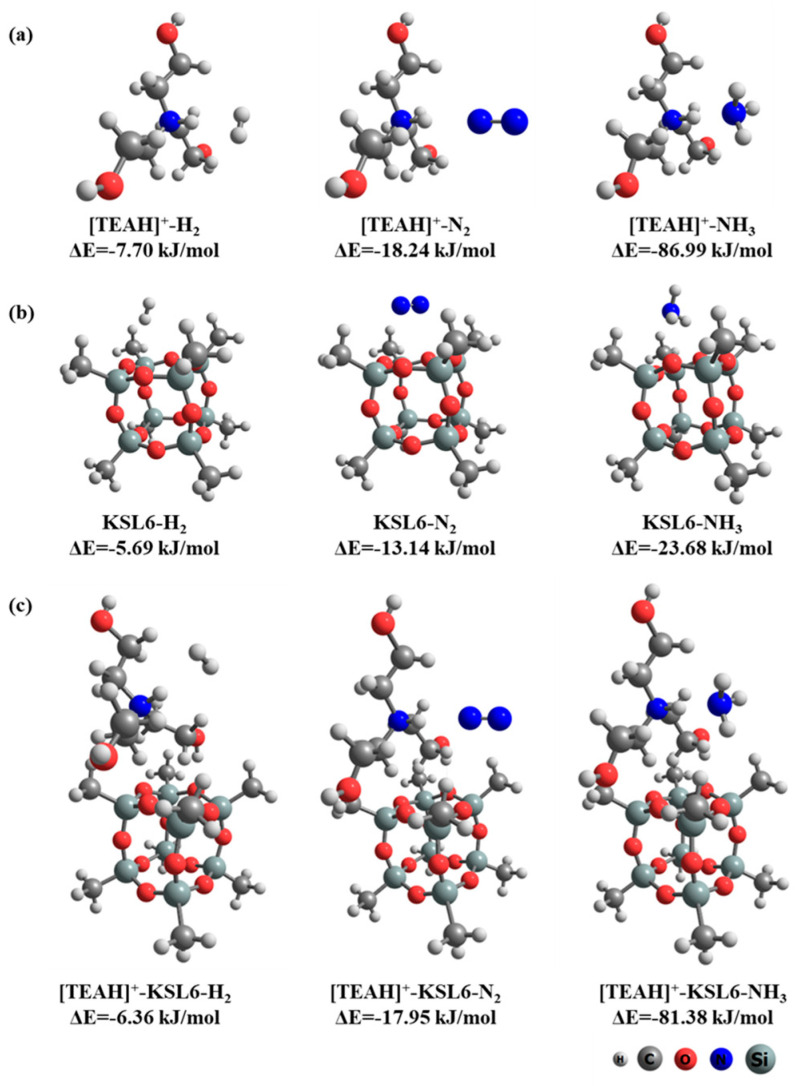
(**a**) Interactions between [TEAH]^+^ and gas molecules. (**b**) Interactions between KSL6 and gas molecules. (**c**) Interactions between [TEAH]^+^-KSL6 composite and gas molecules.

To elucidate the mechanism of high-selective NH_3_/N_2_ and NH_3_/H_2_ separation, DFT calculations were used to investigate the interaction configurations and binding energies of [TEAH]^+^, KSL6, and [TEAH]^+^-KSL6 with H_2_, N_2_, and NH_3_, respectively, as illustrated in Figure 15. The cation [TEAH]^+^ demonstrated strong binding affinity toward NH_3_ with an interaction energy of −86.99 kJ/mol, which was substantially higher than its interactions with N_2_ and H_2_, showing energies of −18.24 and −7.70 kJ/mol, respectively. The aerogel KSL6 structure was modeled as a methyl-terminated cubic cage [62], exhibiting interaction energies of −23.68 kJ/mol for NH_3_, −13.14 kJ/mol for N_2_, and −5.69 kJ/mol for H_2_. When [TEAH]^+^ was incorporated into the KSL6 framework, the composite exhibited an interaction energy of −81.38 kJ/mol with NH_3_, −17.95 kJ/mol with N_2_, and −6.36 kJ/mol with H_2_, demonstrating excellent NH_3_ selectivity. The results suggest that [TEAH]^+^ plays a dominant role in adsorption and separation of NH_3_ for UILACs, further confirming the experimental results.

## 4. Conclusions

A novel design strategy combining HAILs and porous aerogels was proposed and validated for low-concentration NH_3_ capture and separation. The UILACs were successfully synthesized by incorporating HAIL into aerogels. Experimental results demonstrated that at 25 °C and 0.10 MPa, the UILACs achieved a maximum NH_3_ capacity of 164.69 mg NH_3_/g adsorbent and exhibited excellent separation selectivity for NH_3_/H_2_, NH_3_/N_2_, and NH_3_/H_2_/N_2_ gas mixtures at low NH_3_ concentrations. The superior performance of UILACs can be attributed to several key factors:

1. The ultra-low density of aerogels enables high HAIL loading;
2. The multifunctional sites in HAIL enhance NH_3_ molecular adsorption through hydrogen bond interactions;
3. UILACs demonstrated excellent durability and stability over multiple adsorption–desorption cycles.

This work provides new insights into the development of novel porous IL-based adsorbents for gas adsorption and separation applications.

## Figures and Tables

**Table 1:** Thermal stability and pore structures of aerogels and UILACs.

Adsorbents	S_BET_	V_t_	D_p_	T_d_	V_m_/V_t_ Ratio
	(m^2^/g)	(cm^3^/g)	(nm)	(°C)	(%)
KM-W20	446.661	0.6389	3.398	280.78	-
KM-W50	443.899	0.6932	3.400	216.29	-
P200	589.895	2.144	6.552	580.77	-
P250F	713.004	4.0660	16.715	591.60	-
KSL6	716.713	6.1190	12.694	506.92	-
[TEAH][NTf_2_]-P200-90 wt%	1.461	1.143 × 10^−3^	0.751	268.92	46.36
[TEAH][NTf_2_]-P250F-90 wt%	1.798	1.000 × 10^−3^	0.751	270.11	14.97
[TEAH][NTf_2_]-KSL6-90 wt%	3.833	3.356 × 10^−3^	0.718	258.30	25.85

S_BET_, specific surface area; V_t_, total pore volume; D_p_, pore size; V_m_/V_t_ ratio, the proportion of micropore volume to total pore volume; T_d_, thermal decomposition temperature.

**Table 2:** Py-IR analysis of aerogels and UILACs.

Adsorbents	B Acid	L Acid	Total Acid	NH_3_ Capacity
	(μmol/g)	(μmol/g)	(μmol/g)	(mg/g)
P200	-	71.67	71.67	47.429
P250F	-	63.12	63.12	42.537
KSL6	-	51.21	51.21	24.849
[TEAH][NTf_2_]-P200-90 wt%	97.30	90.64	187.94	164.69
[TEAH][NTf_2_]-P250F-90 wt%	79.88	75.37	153.55	155.09
[TEAH][NTf_2_]-KSL6-90 wt%	86.29	70.24	150.53	160.09

B acid, Brønsted acid content; L acid, Lewis acid content; total acid, Brønsted acid and Lewis acid content; NH_3_ capacity, NH_3_ static capacity at 25 °C and 0.10 MPa.

## Data Availability

All experimental data and results presented in this study are included in the main text and its Supplementary Materials. No external datasets were used.

## Supplementary Materials

The following Supplementary Materials can be downloaded at https://www.mdpi.com/article/10.3390/nano15070526/s1, including physicochemical properties and absorption performance of HAILs; XRD and TGA curves of HAILs and UILACs; SEM and TEM images of P200 and P250F series and their breakthrough tests; and related calculation formulas (Figures S1–S20, Tables S1–S7, and Equations (S1)–(S3)).
