# Supplementary material for: Ultramicroporous Ionic Liquid-Supported Aerogel Composites
Source: Nanomaterials (Basel). 2025 Mar 31;15(7):526. doi: 10.3390/nano15070526 (PMC11990487; doi:10.3390/nano15070526)
Supplement: Supplementary file 1 [file nanomaterials-15-00526-s001.zip › nanomaterials-3525228-supplementary.pdf]

Supplementary Materials

Ultramicroporous Ionic Liquid-supported Aerogel Composites for Trace Ammonia Separation

Wenshuo Pan[a,b], Shaojuan Zeng[b*], Jiang Chang[b], Guilin Li[b], Wei Zhang[a], Xiangping Zhang[c]

[a] College of New Material and Chemical Engineering, Beijing Institute of Petrochemical Technology, 102617, Beijing, China

[b] Beijing Key Laboratory of Solid State Battery and Energy Storage Process, CAS Key Laboratory of Green Process and Engineering, State Key Laboratory of Mesoscience and Engineering, Institute of Process Engineering, Chinese Academy of Sciences, 100190, Beijing, China

[c] College of Chemical Engineering and Environment, China University of Petroleum, 102249, Beijing, China

---

[*]Corresponding author. Tel./fax: +86-010-82544875.

E-mail addresses: sjzeng@ipe.ac.cn (S. J. Zeng), No.1, North 2nd Street, Zhongguancun, Haidian District, Beijing, 100190, China.

1. Ideal Adsorbed Solution Theory Equation:

$$S=(X_1/Y_1)/(X_2/Y_2) \qquad \textbf{(Equation S1)}$$

S: selectivity coefficient for the separation of component 1 from component 2;

X_1: mole fraction of component 1 in the adsorbed phase;

Y_1: mole fraction of component 1 in the gas phase;

X_2: mole fraction of component 2 in the adsorbed phase;

Y_2: mole fraction of component 2 in the gas phase.

2. Freundlich Model Equation:

$$q = KP^{\frac{1}{m}} \qquad \textbf{(Equation S2)}$$

q: equilibrium adsorption capacity (mg NH_3/g adsorbent);

P: equilibrium pressure (bar);

K: Freundlich constant (L/g);

m: Freundlich constant (dimensionless).

3. Clausius-Clapeyron Equation：

$$Q_{st} = R\left[\frac{\partial lnP}{\partial\frac{1}{T}}\right]_{q_e} \qquad \textbf{(Equation S3)}$$

Q_{st}: isosteric heat of adsorption (kJ/mol);

T: adsorption temperature (K);

q_e: adsorption capacity at given T and P (mg NH_3/g adsorbent);

P: equilibrium pressure (bar);

R: gas constant (8.314×10^{-3} kJ/mol·K).

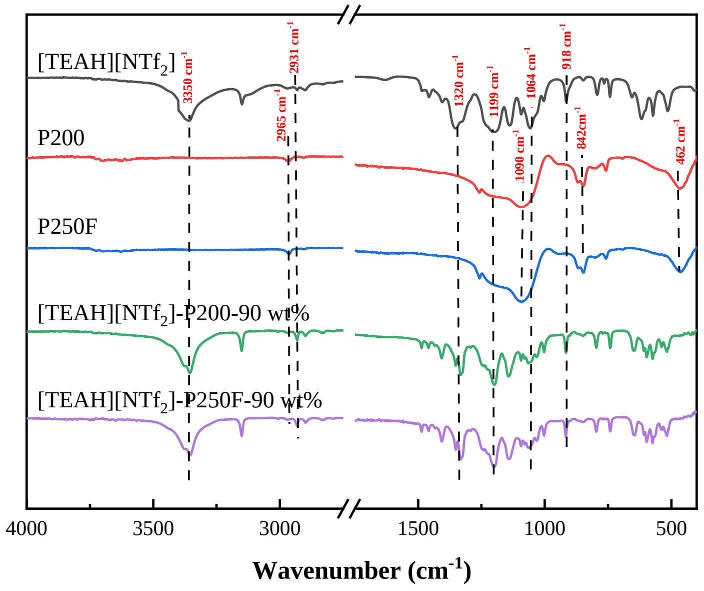

Figure S1. FT-IR spectra of [TEAH][NTf2], P200, P250F, [TEAH][NTf2]-P200-90 wt% and [TEAH][NTf2]-P250F-90 wt%.

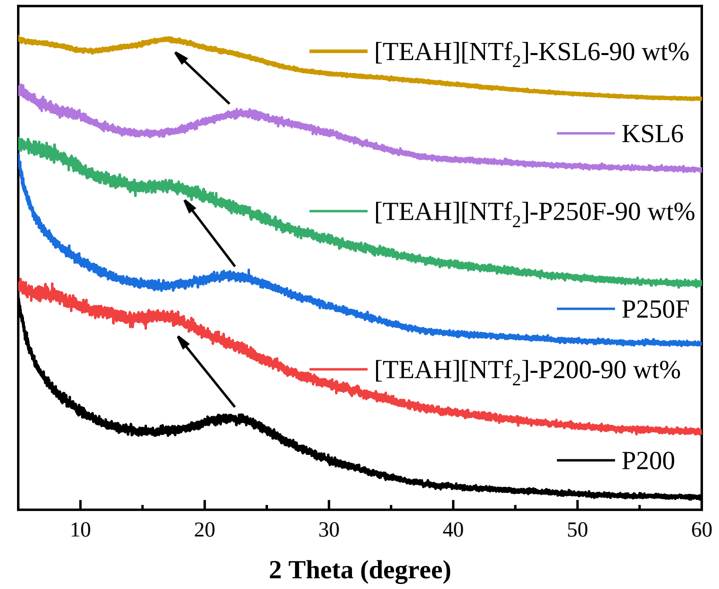

Figure S2. XRD patterns of pure aerogels and their corresponding UILACs.

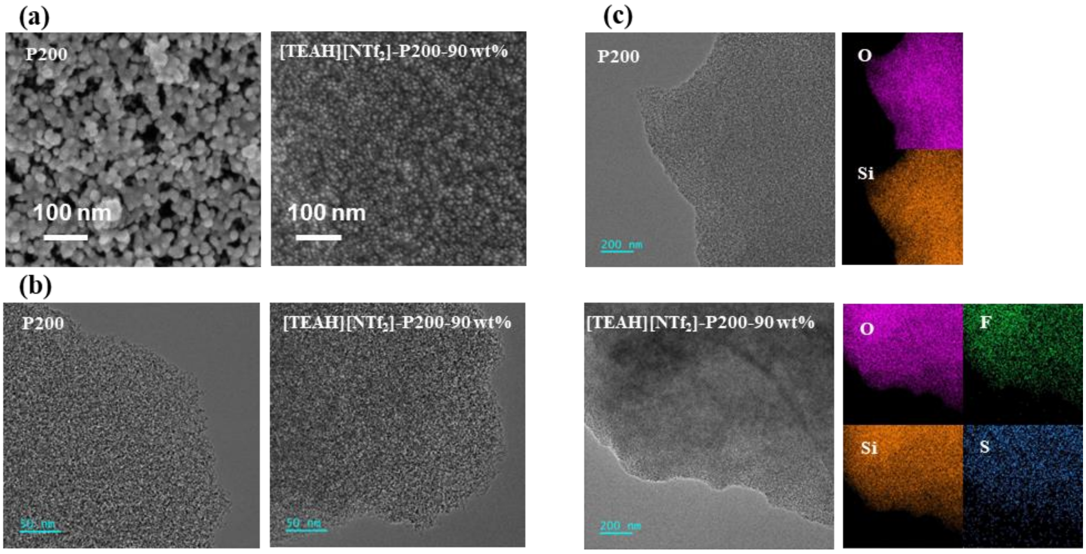

Figure S3. (a) SEM images, (b) TEM images, and (c) mapping images of P200 and [TEAH][NTf_2]-P200-90 wt%.

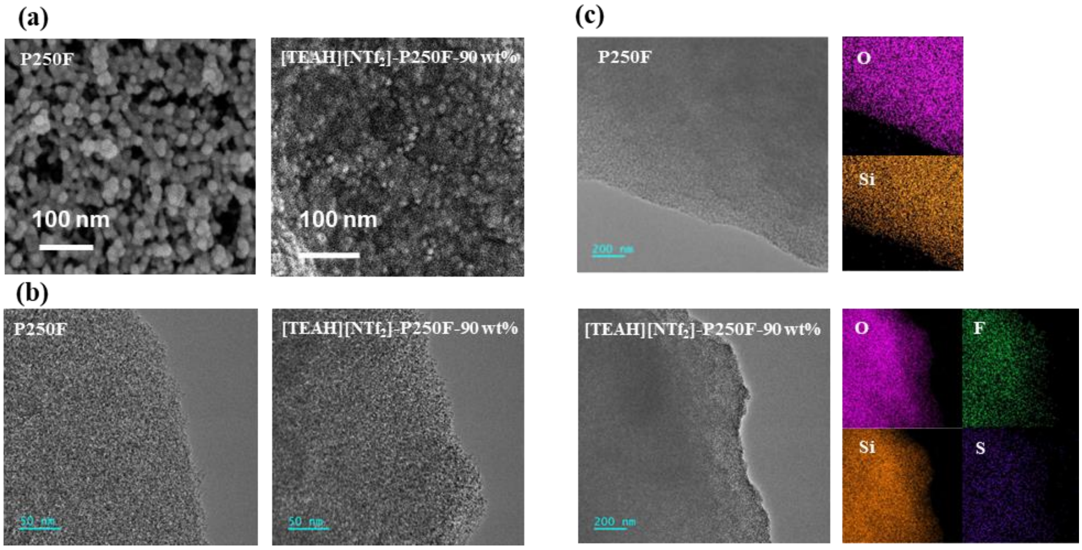

Figure S4. (a) SEM images, (b) TEM images, and (c) mapping images of P250F and [TEAH][NTf_2]-P250F-90 wt%.

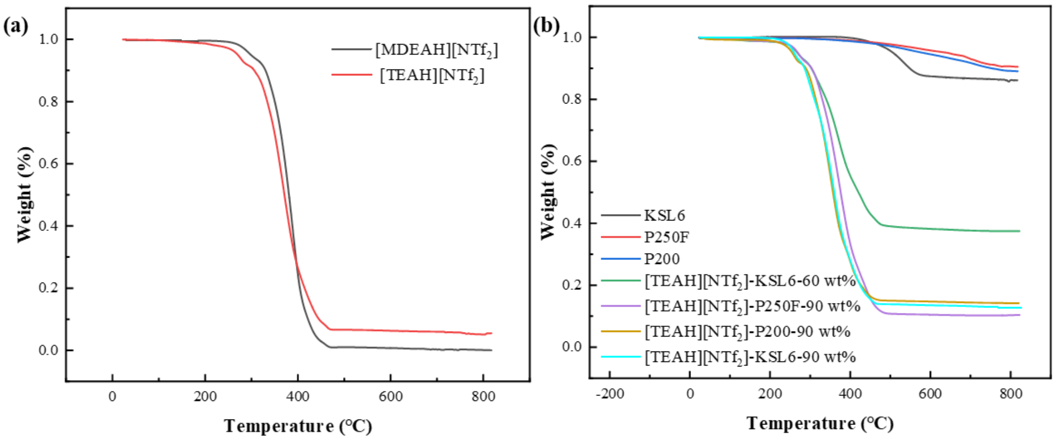

Figure S5. TGA curves: (a) thermal decomposition profiles of HAILs and (b) thermal stability of aerogels and UILACs.

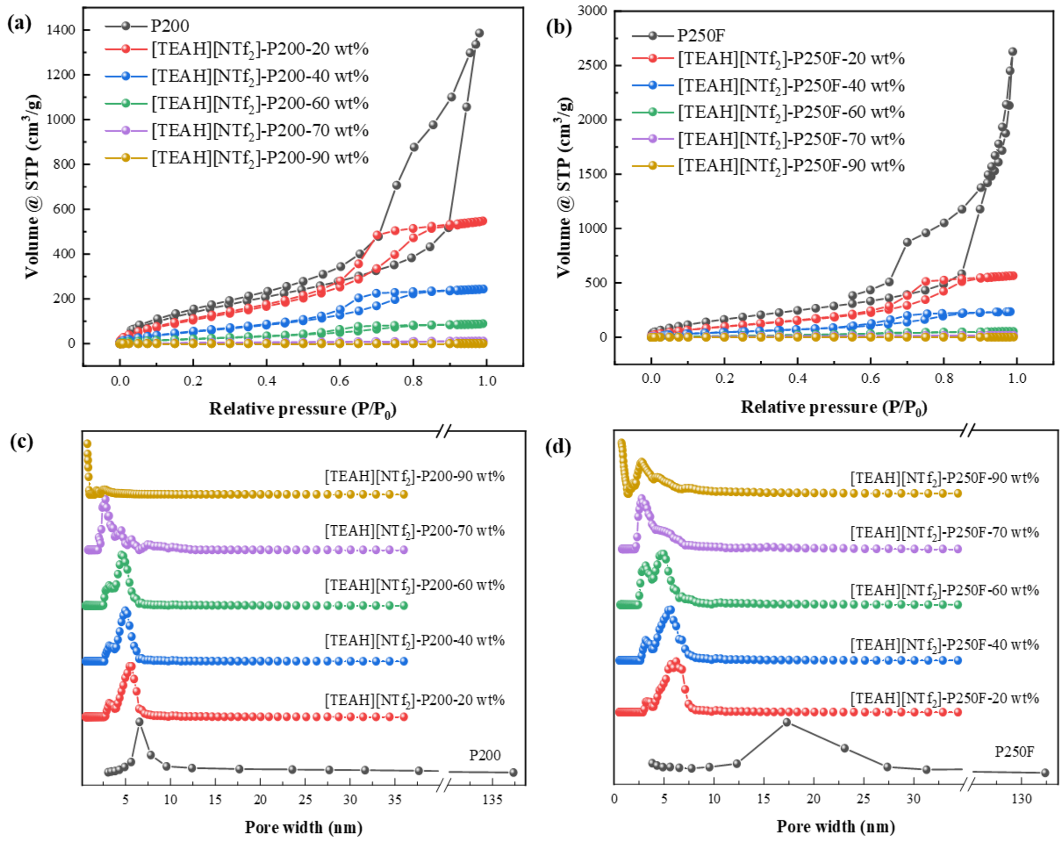

Figure S6. N_2 adsorption-desorption isotherms of (a) [TEAH][NTf2]-P200 composites, (b) [TEAH][NTf2]-P250F composites; Pore size distributions of (c) [TEAH][NTf2]-P200 composites, (d) [TEAH][NTf2]-P250F composites.

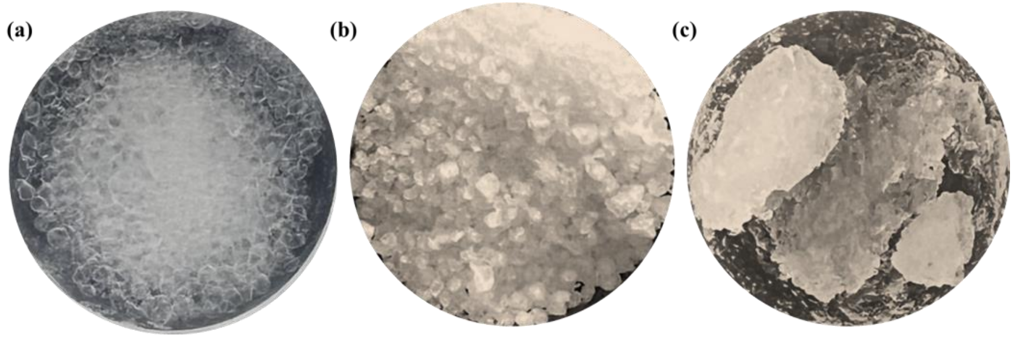

Figure S7. Pictures of (a) P200; (b) [TEAH][NTf_2]-P200-90 wt% and (c) [TEAH][NTf_2]-P200-91 wt%.

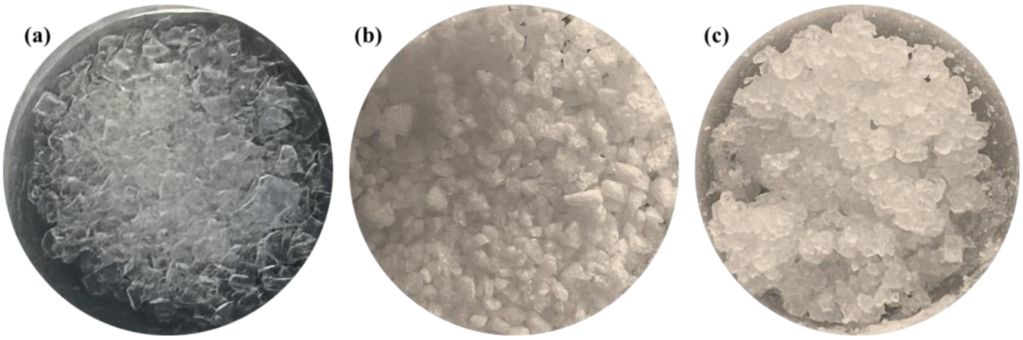

Figure S8. Pictures of (a) P250F; (b) [TEAH][NTf_2]-P250F-90 wt% and (c) [TEAH][NTf_2]-P250F-91 wt%.

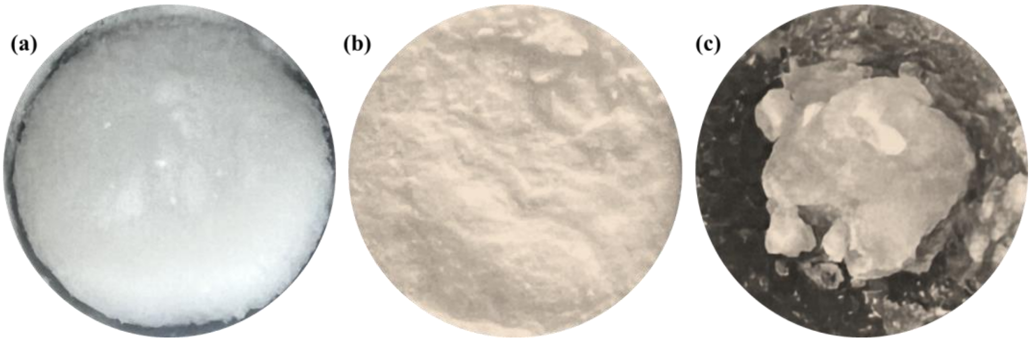

Figure S9. Pictures of (a) KSL6; (b) [TEAH][NTf_2]-KSL6-90 wt% and (c) [TEAH][NTf_2]-KSL6-91 wt%.

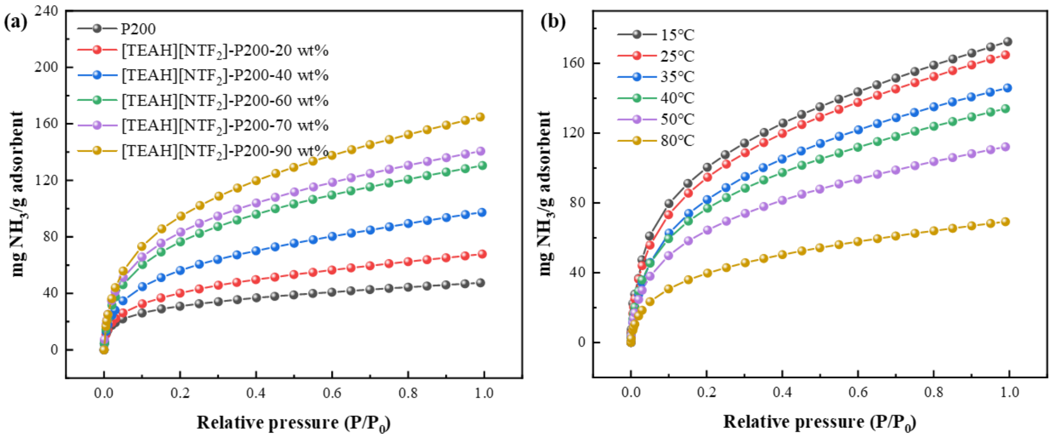

Figure S10. NH_3 capacity of (a) P200 with different HAIL loadings and (b) [TEAH][NTf$_2$]-P200-90 wt% at different temperatures.

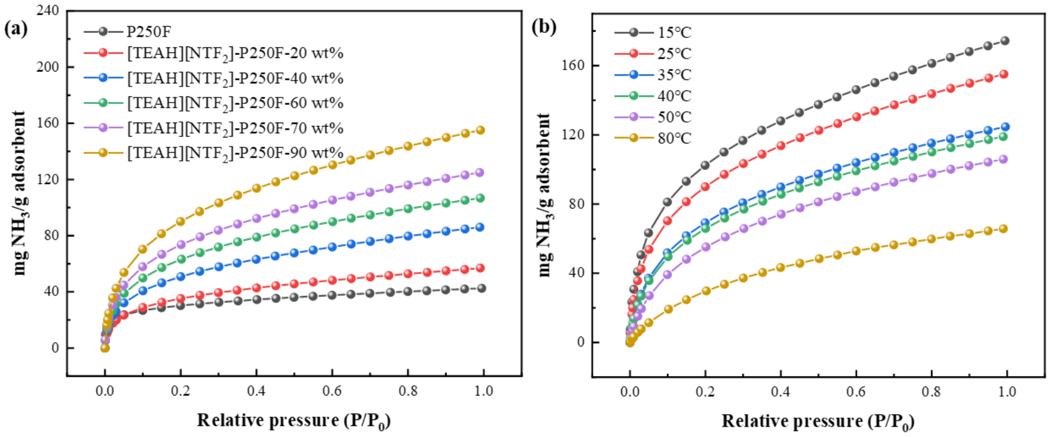

Figure S11. NH_3 capacity of (a) P250F with different HAIL loadings and (b) [TEAH][NTf$_2$]-P250F-90 wt% at different temperatures.

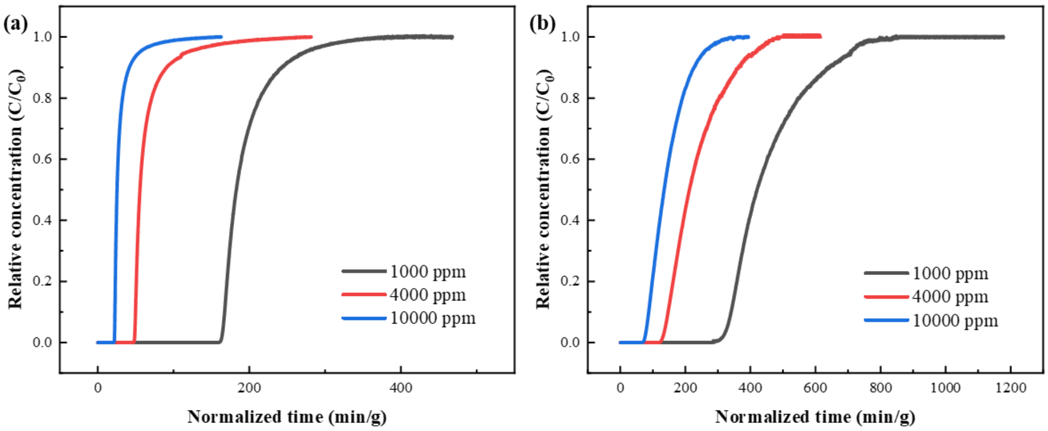

Figure S12. Breakthrough curves of NH_3/H_2 binary mixtures at different NH_3 concentrations: (a) P200 and (b) [TEAH][NTf$_2$]-P200-90 wt%.

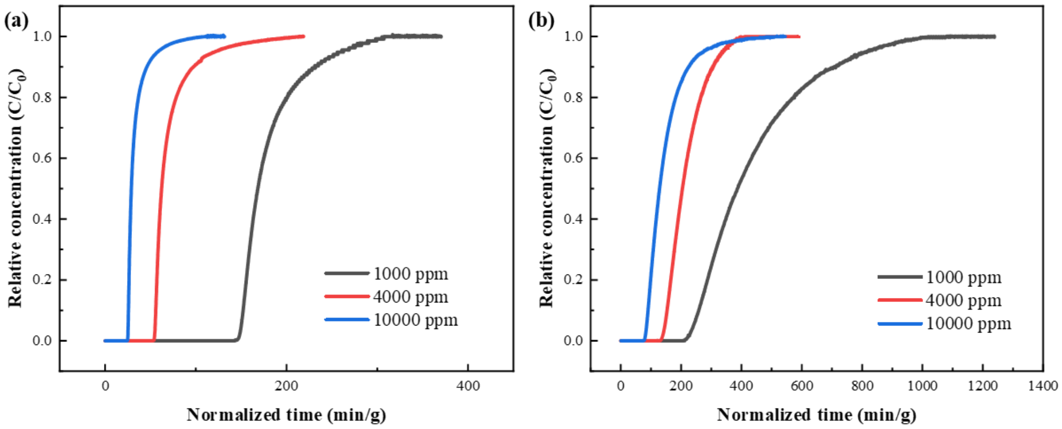

Figure S13. Breakthrough curves of NH_3/H_2 binary mixtures at different NH_3 concentrations: (a) P250F and (b) [TEAH][NTf$_2$]-P250F-90 wt%.

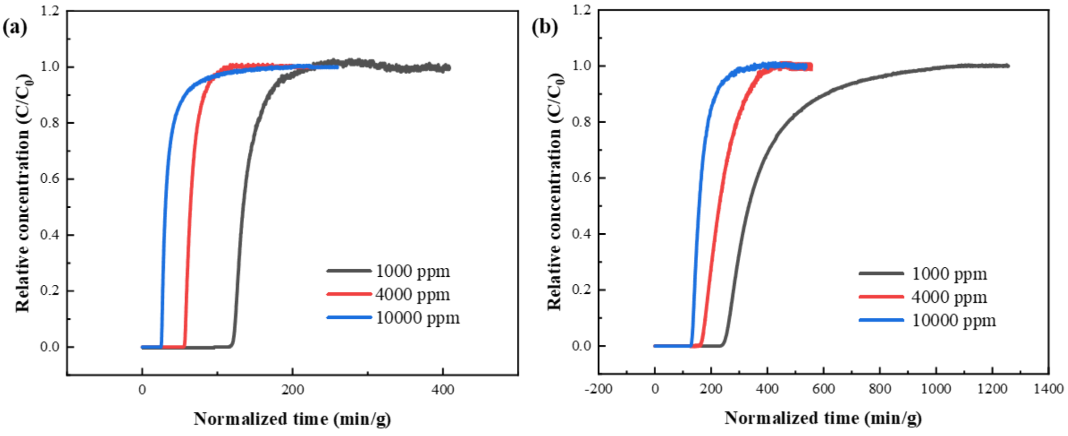

Figure S14. Breakthrough curves of NH_3/N_2 binary mixtures at different NH_3 concentrations: (a) P200 and (b) [TEAH][NTf2]-P200-90 wt%.

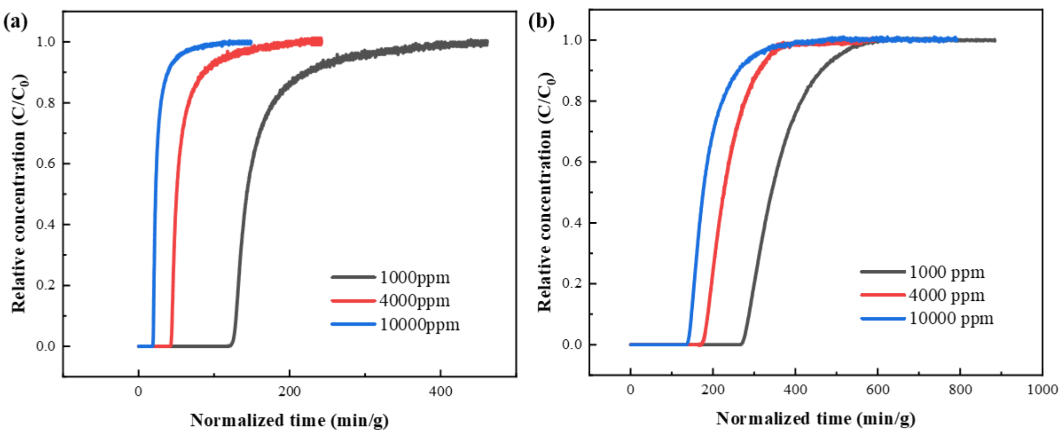

Figure S15. Breakthrough curves of NH_3/N_2 binary mixtures at different NH_3 concentrations: (a) P250F and (b) [TEAH][NTf2]-P250F-90 wt%.

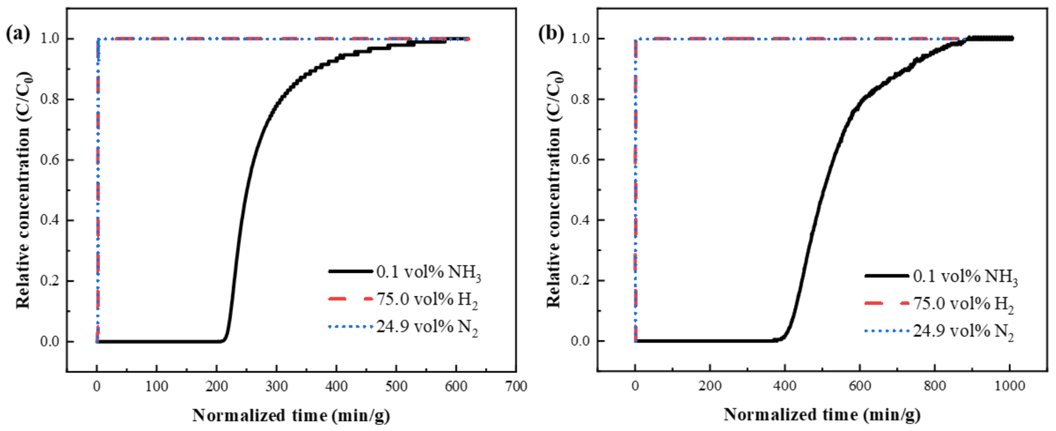

Figure S16. Breakthrough curves for $NH_3/N_2/H_2$ (0.1 vol%/24.9 vol%/75.0 vol%) ternary mixtures through different materials: (a) P200 and (b) [TEAH][NTf_2]-P200-90 wt%.

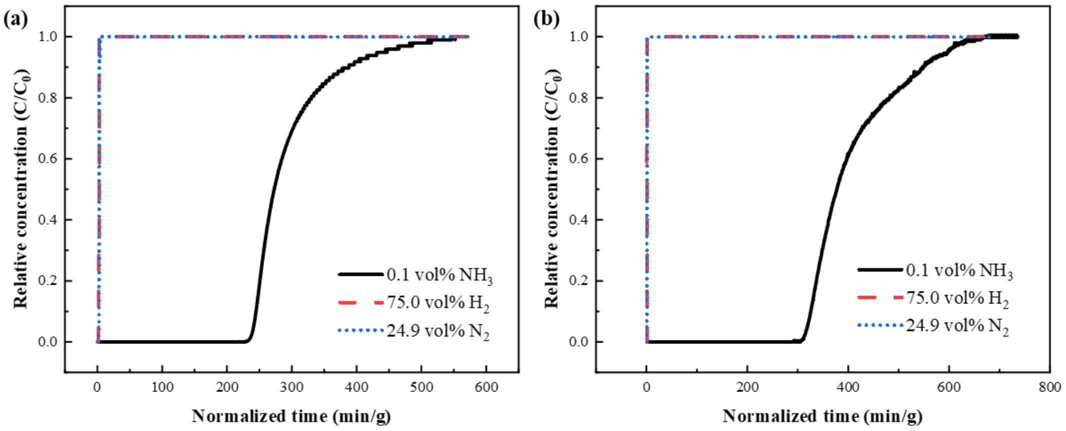

Figure S17. Breakthrough curves for $NH_3/N_2/H_2$ (0.1 vol%/24.9 vol%/75.0 vol%) ternary mixtures through different materials: (a) P250F and (b) [TEAH][NTf_2]-P250F-90 wt%.

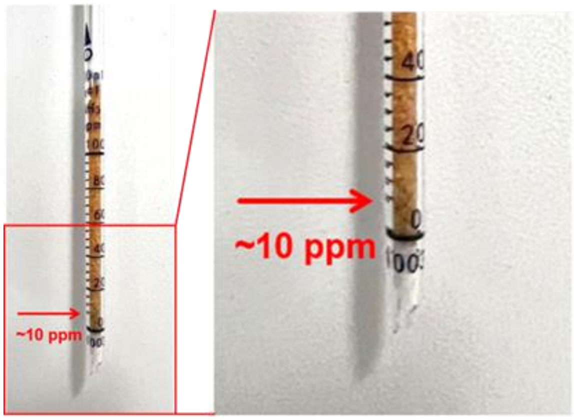

Figure S18. Picture of gas detection tube after testing outlet gas.

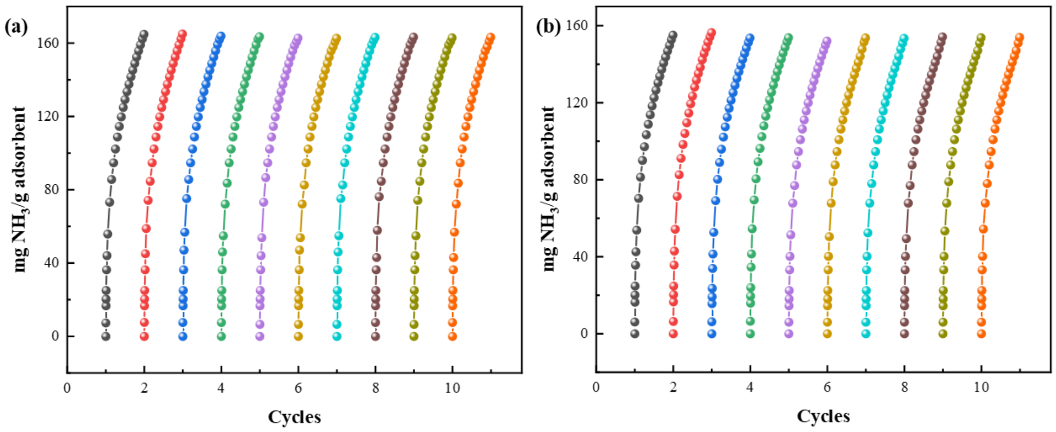

Figure S19. 10 cycles NH_3 capacity of (a) [TEAH][NTf$_2$]-P200-90 wt% and (b) [TEAH][NTf$_2$]-P250F-90 wt%.

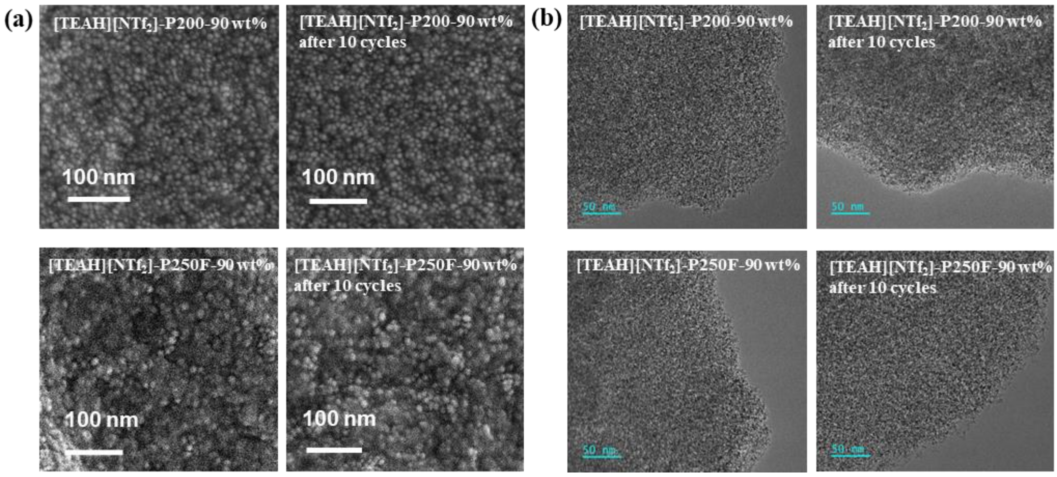

Figure S20. Characterization of (a) SEM images and (b) TEM images before and after 10 cycles.

Table S1. Properties of HAILs.

HAILs	Water content (wt%)	Temperature (°C)	Density (g/cm^3)	Viscosity (mPa·s)
[MDEAH][NTf_2]	0.0736	20	1.600	546.52
		30	1.590	168.15
		40	1.580	88.795
		50	1.569	79.302
		60	1.559	50.193
		70	1.549	33.965
[TEAH][NTf_2]	0.1443	20	1.590	872.55
		30	1.581	382.05
		40	1.572	190.06
		50	1.563	106.17
		60	1.554	64.846
		70	1.544	42.300

Table S2. Aerogels features were provided by the manufacturer.

Aerogels	P200	P250F	KSL6	KM-W20	KM-W50
Particle size (mm)	0.1-1.2	0.5-4.0	0.01-1	~0.02	~0.05
Pore diameter (nm)	~20	~20	-	~15	~15
Porosity	>90%	>90%	-	>90%	>90%
Particle density (kg/m^3)	120-180	120-180	<100	-	-
Bulk density (kg/m^3)	75-59	65-85	-	60-120	60-120
Hydrophobic	√	√	√	√	√
Surface area (m^2/g)	600-800	600-800	-	400-700	400-700
CAS RN	102262-30-6	102262-30-6	-	-	-

Table S3. Fitting parameters of Freundlich model for KSL6 and [TEAH][NTf_2]-KSL6-90 wt% under different temperatures.

Adsorbents	Temperature (°C)	Parameter		R^2
		K	m	
KSL6	15	33.430	2.374	0.991
	25	22.781	3.031	0.992
	35	19.502	2.378	0.991
[TEAH][NTf_2]-KSL6-90 wt%	15	30.154	2.699	0.997
	25	22.781	3.031	0.997
	35	18.668	2.703	0.996

Table S4. Breakthrough data of aerogels and UILACs under binary NH_3/H_2 mixture of at 25°C.

NH_3/H_2 (ppm)	NH_3 capacity (mg/g)	NH_3/H_2 selectivity (mol/mol)	Breakthrough point (min/g)	NH_3 capacity (mg/g)	NH_3/H_2 selectivity (mol/mol)	Breakthrough point (min/g)
		P200			[TEAH][NTf2]-P200-90 wt%	
1000	4.959	201	162.996	7.464	1924	312.762
4000	6.671	89	48.635	14.292	1065	128.430
10000	7.704	103	22.032	22.364	1748	73.179
		P250F			[TEAH][NTf2]-P250F-90 wt%	
1000	4.803	142	148.149	6.632	1631	217.362
4000	6.691	85	54.522	13.215	898	137.067
10000	7.597	79	25.120	22.306	1267	79.997
		KSL6			[TEAH][NTf2]-KSL6-90 wt%	
1000	2.972	78	121.601	8.937	2460	374.059
4000	5.027	41	56.131	13.345	1199	159.128
10000	5.732	45	19.630	23.931	1250	127.522

Table S5. Breakthrough data of aerogels and UILACs under binary NH_3/N_2 mixture of at 25°C.

NH_3/N_2 (ppm)	NH_3 capacity (mg/g)	NH_3/N_2 selectivity (mol/mol)	Breakthrough point (min/g)	NH_3 capacity (mg/g)	NH_3/N_2 selectivity (mol/mol)	Breakthrough point (min/g)
		P200			[TEAH][NTf2]-P200-90 wt%	
1000	4.791	656	120.596	7.097	8907	246.151
4000	7.820	268	61.677	14.567	4638	166.056
10000	11.791	179	20.654	22.473	3160	130.741
		P250F			[TEAH][NTf2]-P250F-90 wt%	
1000	4.499	567	124.154	7.164	8152	270.156
4000	8.423	284	48.832	14.722	4523	173..978
10000	9.499	143	26.548	23.130	3117	87.484
		KSL6			[TEAH][NTf2]-KSL6-90 wt%	
1000	3.357	473	160.486	8.587	10474	374.567
4000	5.423	211	49.203	16.537	6018	288.205
10000	11.357	220	23.517	24.012	3544	126.975

Table S6. Breakthrough data of aerogels and UILACs under ternary gas mixture of $NH_3/N_2/H_2$ (0.1 vol%/24.9 vol%/75.0 vol%) at 25°C.

Adsorbents	NH_3 breakthrough point (min/g)	NH_3 capacity (mg/g)	NH_3/H_2 selectivity	NH_3/N_2 selectivity
P200	215.945	4.136	185	221
P250F	236.243	4.455	149	168
KSL6	146.337	3.263	102	124
[TEAH][NTf$_2$]-P200-90 wt%	394.510	6.174	1918	6699
[TEAH][NTf$_2$]-P250F-90 wt%	312.728	6.169	2110	7398
[TEAH][NTf$_2$]-KSL6-90 wt%	444.035	7.666	2141	8061

Table S7. Comparison of NH_3 capacity and selectivity.

Absorbents	Temperature (°C)	NH_3 partial pressure (MPa)	NH_3 capacity (mg/g)	NH_3 selectivity	Ref.
SBA/3.0 wt% [MimC$_3$SO$_3$H][TfO]	25	0.10	130.9	-	[1]
NPC-700-[C$_3$N][SO$_4$H]	25	0.10	200.6	-	[2]
20 wt%[2-Mim] [NTf$_2$]@AC-980	30	0.10	68.61	-	[3]
49 wt% [Bmim]$_2$[Co (NCS)$_4$]@SG	30	0.10	99.808	-	[4]
[BOHmim][Zn$_2$Cl$_5$] @MIL-101(Cr)	25	0.10	410.0	-	[5]
50 wt%[TEAH] [CF$_3$SO$_3$]@MCM41	40	0.10	114.3	-	[6]
[TEAH][NTf$_2$]-P200-90 wt%	25	0.10	164.49	-	This work
[TEAH][NTf$_2$]-P250F-90 wt%	25	0.10	155.09	-	This work
[TEAH][NTf$_2$]-KSL6-90 wt%	25	0.10	160.09	-	This work
LiCl@MIL-53-(OH)$_2$-43.4	25	0.02	472.6	-	[7]
60 wt % [EdteH$_6$][TfO]$_2$@HZ60	40	0.10	116.15	-	[8]
NPC-600-[C$_3$N][SO$_4$H]	25	0.01	-	427.7[a]	[2]
NPC-600-[C$_3$N][SO$_4$H]	25	0.01	-	502.2[b]	[2]
LiLSX	20	0.0017	92.7[b]	-	[9]
[C$_8$C$_1$Im]Cl/ CuCl$_2$	30	0.01	27.14[b]	-	[10]
LiCl@MIL-53-(OH)$_2$-43.4	25	0.004	-	3571[b]	[7]
60 wt % [EdteH$_6$][TfO]$_2$@HZ60	40	-	-	468.0[b]	[8]
[TEAH][NTf$_2$]-P200-90 wt%	25	0.001	7.464[a]	1924[a]	This work
[TEAH][NTf$_2$]-P250F-90 wt%	25	0.001	6.632[a]	1631[a]	This work
[TEAH][NTf$_2$]-KSL6-90 wt%	25	0.001	8.937[a]	2459[a]	This work

[TEAH][NTf2]-P200-90 wt%	25	0.001	7.097[b]	8907[b]	This work
[TEAH][NTf2]-P250F-90 wt%	25	0.001	7.164[b]	8152[b]	This work
[TEAH][NTf2]-KSL6-90 wt%	25	0.001	8.587[b]	10474[b]	This work

a: data under NH_3/H_2 mixture, b: data under NH_3/N_2 mixture.

Reference

1. Ruckart, K.N.; Zhang, Y.; Reichert, W.M.; Peterson, G.W.; Glover, T.G. Sorption of Ammonia in Mesoporous-Silica Ionic Liquid Composites. *Ind. Eng. Chem. Res.* **2016**, *55*, 12191–12204, doi:10.1021/acs.iecr.6b02041.
2. Zhu, Q.; Zhang, W.; Zhong, S.; Xiao, Y.; Qian, H.; Zheng, A.; Liu, F.; Jiang, L. Precisely Capture Trace Ammonia from Fuel Cell System over Ionic Liquid Grafted Hierarchically Porous Carbons. *Chem. Eng. J.* **2024**, *483*, 149349, doi:10.1016/j.cej.2024.149349.
3. Yu, M.; Zeng, S.; Wang, Z.; Hu, Z.; Dong, H.; Nie, Y.; Ren, B.; Zhang, X. Protic Ionic-Liquid-Supported Activated Carbon with Hierarchical Pores for Efficient NH_3 Adsorption. *ACS Sustain. Chem. Eng.* **2019**, *7*, 11769–11777, doi:10.1021/acssuschemeng.9b02051.
4. Zeng, S.; Wang, J.; Li, P.; Dong, H.; Wang, H.; Zhang, X.; Zhang, X. Efficient Adsorption of Ammonia by Incorporation of Metal Ionic Liquids into Silica Gels as Mesoporous Composites. *Chem. Eng. J.* **2019**, *370*, 81–88, doi:10.1016/j.cej.2019.03.180.
5. Han, G.; Liu, C.; Yang, Q.; Liu, D.; Zhong, C. Construction of Stable IL@MOF Composite with Multiple Adsorption Sites for Efficient Ammonia Capture from Dry and Humid Conditions. *Chem. Eng. J.* **2020**, *401*, 126106, doi:10.1016/j.cej.2020.126106.
6. Zheng, S.; Xu, Q.; Zeng, S.; Li, G.; Jiang, H.; Sun, X.; Zhang, X. Porous Multi-Site Ionic Liquid Composites for Superior Selective and Reversible Adsorption of Ammonia. *Sep. Purif. Technol.* **2023**, *310*, 123161, doi:10.1016/j.seppur.2023.123161.
7. Shi, Y.; Wang, Z.; Li, Z.; Wang, H.; Xiong, D.; Qiu, J.; Tian, X.; Feng, G.; Wang, J. Anchoring LiCl in the Nanopores of Metal–Organic Frameworks for Ultra-High Uptake and Selective Separation of Ammonia. *Angew. Chem. Int. Ed.* **2022**, *61*, e202212032, doi:10.1002/anie.202212032.
8. Li, Y.; Zeng, S.; Zheng, S.; Zhao, T.; Sun, X.; Bai, L.; Deng, C.; Zhang, X. Mesoporous Multiproton Ionic Liquid Hybrid Adsorbents for Facilitating NH_3 Separation. *Ind. Eng. Chem. Res.* **2023**, *62*, 2829–2842, doi:10.1021/acs.iecr.2c03193.
9. Ouyang, W.; Zheng, S.; Wu, C.; Hu, X.; Chen, R.; Zhuo, L.; Wang, Z. Dynamic Ammonia Adsorption by FAU Zeolites to below 0.1 ppm for Hydrogen Energy

Applications. *Int. J. Hydrog. Energy* **2021**, *46*, 32559–32569, doi:10.1016/j.ijhydene.2021.07.107.

10. Kohler, F.T.U.; Popp, S.; Klefer, H.; Eckle, I.; Schrage, C.; Böhringer, B.; Roth, D.; Haumann, M.; Wasserscheid, P. Supported Ionic Liquid Phase (SILP) Materials for Removal of Hazardous Gas Compounds – Efficient and Irreversible NH_3 Adsorption. *Green Chem.* **2014**, *16*, 3560, doi:10.1039/c3gc42275e.
